# Colchicine as an Adjunctive Therapy to Improve Ischemic Stroke Reperfusion Outcomes in Mice

**DOI:** 10.1002/cns.70922

**Published:** 2026-07-09

**Authors:** Danni Wang, Yuwen Xiu, Mengxuan Shi, Yingjie Wang, Di Zhou, Mitchell D. Kilgore, Lauren Dumont, Thin Yadanar Sein, Yinghua Jiang, Nicole Kazour, Ning Liu, Aaron S. Dumont, Qiang Liu, Xiaoying Wang

**Affiliations:** ^1^ Department of Neurology, Tianjin Neurological Institute Tianjin Medical University General Hospital Tianjin China; ^2^ Department of Neurosurgery and Neurology, Clinical Neuroscience Research Center Tulane University School of Medicine New Orleans Louisiana USA

**Keywords:** acute ischemic stroke, blood–brain barrier, colchicine, mechanical thrombectomy, neutrophil extracellular trap, thrombo‐inflammation

## Abstract

**Background:**

Mechanical thrombectomy (MT) has become a cornerstone therapy for acute ischemic stroke (AIS) caused by large‐vessel occlusion; however, incomplete microvascular reperfusion and blood–brain barrier (BBB) disruption frequently limit its clinical benefit. Emerging evidence implicates neutrophil‐driven thrombo‐inflammation, particularly neutrophil extracellular trap (NET)–mediated microvascular obstruction, as a key mechanism underlying post‐reperfusion hypoperfusion and secondary neurovascular injury. Colchicine, an FDA‐approved microtubule inhibitor, potently suppresses neutrophil effector functions with a favorable safety profile, making it a promising adjunct to ischemia–reperfusion.

**Methods:**

Using a transient middle cerebral artery occlusion (tMCAO) mouse model of ischemia–reperfusion, colchicine (0.8 mg/kg, intraperitoneal) was administered at reperfusion. We assessed circulating neutrophil response and activation, neutrophil F‐actin polymerization, NET formation, microvascular fibrin(ogen) deposition, cerebral blood flow, leukocyte brain infiltration, BBB integrity, and neuroinflammation at acute and subacute time points. Whole‐blood phagocytosis and bone marrow cellularity were examined at Day 3 after tMCAO. Neutrophil depletion was achieved using an anti‐Ly6G antibody. Long‐term neurological outcomes and brain tissue loss were evaluated up to 14 days after tMCAO.

**Results:**

Colchicine administration at reperfusion significantly blunted early neutrophil increase and activation, inhibited neutrophil F‐actin polymerization, and suppressed intravascular NET formation and microvascular fibrin(ogen) deposition in tMCAO mice. It also improved cerebral blood flow, reduced leukocyte brain infiltration, attenuated BBB disruption, and decreased infarct size. Importantly, colchicine‐treated mice exhibited sustained improvements in sensorimotor and cognitive function and reduced chronic brain tissue loss. Notably, colchicine did not exacerbate post‐stroke immunosuppression, and its neuroprotective effects were abolished by neutrophil depletion.

**Conclusions:**

Colchicine administered at reperfusion suppresses neutrophil‐driven thrombo‐inflammation, preserves BBB integrity, and improves cerebral blood flow, resulting in reduced ischemic brain injury and long‐term neurological deficits after ischemic stroke. These findings suggest colchicine might be developed as a potential adjunctive therapy to limit reperfusion‐associated microvascular dysfunction and neurovascular injury.

## Graphical abstract

In a tMCAO mouse model of ischemia–reperfusion, colchicine administered at reperfusion suppresses neutrophil activation by inhibiting F‐actin polymerization. Thereby, it improves cerebral blood flow by limiting NET formation and intravascular fibrin(ogen) deposition and preserves BBB integrity by attenuating cerebrovascular inflammation and neuroinflammation, ultimately improving neurological outcomes.

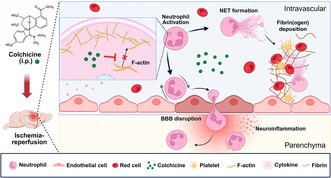

## 1 Introduction

Acute ischemic stroke (AIS) remains one of the leading causes of mortality and long‐term disability worldwide [1]. Early recanalization and reperfusion are cornerstones of therapy for AIS [2, 3]. In addition to intravenous recombinant tissue plasminogen activator (rt‐PA), mechanical thrombectomy (MT) is now widely used to rapidly restore cerebral blood flow in patients with acute large‐vessel occlusion [3, 4, 5]. However, about 50% of patients treated with MT still experience poor functional recovery and unfavorable outcomes [6, 7]. Critically, the successful reopening of an occluded large artery does not ensure restoration of effective microvascular perfusion. A substantial proportion of reperfused brains exhibit persistent capillary venular obstruction (hypoperfusion), accompanied by blood–brain barrier (BBB) disruption, edema, and hemorrhagic transformation, which together limit neurological recovery despite macrovascular recanalization [8, 9, 10]. Accumulating experimental and clinical investigations have demonstrated that one of the key pathological contributors to the shortcomings of MT reperfusion therapy is the rapid, neutrophil‐derived thrombo‐inflammatory processes in the cerebral microcirculation during early reperfusion [11, 12, 13].

Neutrophils are increasingly recognized as a central upstream driver of microvascular failure following cerebral ischemia–reperfusion [14, 15]. Rapidly recruited and activated in the circulation within minutes after reperfusion, neutrophils can simultaneously orchestrate the key pathological processes underlying microvascular no‐reflow, hypoperfusion, and BBB injury [16]. Upon activation, neutrophils undergo cytoskeletal remodeling that increases cellular stiffness and promotes adhesion within capillaries and post‐capillary venules, directly impeding red blood cell transit and elevating microvascular resistance [17, 18]. In parallel, neutrophils amplify flow collapse by recruiting platelets, inducing endothelial contraction, and promoting perivascular swelling [19, 20]. Neutrophil degranulation and NET formation further drive thrombo‐inflammation by providing a procoagulant scaffold for fibrin deposition and platelet‐rich microthrombus formation, whereas neutrophil‐derived proteases, reactive oxygen species, and NET components damage endothelial junctions and the glycocalyx, leading to BBB disruption [21, 22]. Together, these events establish a self‐reinforcing loop linking thrombosis, hypoperfusion, and vascular injury. Because large‐vessel recanalization with mechanical thrombectomy does not ensure restoration of microvascular perfusion, these observations highlight neutrophils as a compelling therapeutic target [14]. It has been recognized that adjunctive strategies aimed at restraining neutrophil activation and NET formation can convert macrovascular recanalization into effective tissue reperfusion, limit BBB disruption, and improve neurological outcomes after ischemic stroke [23, 24].

Colchicine, an FDA‐approved microtubule inhibitor, has long been used as an anti‐inflammatory agent across diverse neutrophil activation‐related inflammatory conditions [25]. Although colchicine shows poor penetration into brain tissue [26], it is relatively selective for neutrophils, largely because it preferentially accumulates in neutrophils that lack the P‐glycoprotein (P‐gp) efflux pump [27]. Colchicine specifically targets a core and shared requirement of neutrophil effector functions: microtubule‐dependent cytoskeletal dynamics [28]. By disrupting microtubule polymerization, colchicine blunts neutrophil polarization/chemotaxis, integrin trafficking and adhesion, granule mobilization (degranulation), and NET release—thereby acting as a mechanistically grounded “cytoskeletal brake” on thrombo‐inflammation [29], identifying neutrophil cytoskeletal regulation as a high‐value therapeutic target to improve the efficacy and safety of current stroke interventions [30]. Importantly, colchicine is clinically available, safe, inexpensive, and translationally feasible, making it attractive as an on‐reperfusion adjunct to enhance microvascular flow and preserve BBB integrity, ultimately improving neurological outcomes.

Recent clinical trials indicate that long‐term low‐dose colchicine confers consistent benefit in secondary prevention of coronary atherosclerotic events [31]. In COLCOT and LoDoCo2, colchicine significantly reduced major adverse cardiovascular events on top of guideline therapy [31, 32], with supportive signals from earlier LoDoCo studies [33, 34]. Although the CONVINCE trial did not demonstrate efficacy in preventing recurrent non‐cardioembolic stroke, it confirmed a broadly reassuring safety profile, with no excess serious adverse events aside from increased gastrointestinal intolerance [35]. Together, these data support colchicine as a safe, clinically tractable candidate for short‐term, adjunctive modulation of thrombo‐inflammation during ischemia–reperfusion therapy.

We therefore hypothesize that colchicine may act as a cytoskeletal brake on neutrophil activation, providing a mechanistically grounded and translationally feasible strategy to mitigate NET‐mediated cerebrovascular thrombo‐inflammation after reperfusion therapy. To test this hypothesis, we used a tMCAO mouse model of ischemia–reperfusion to investigate the effects of colchicine on neutrophil F‐actin polymerization, inflammatory activation, NET formation, cerebral blood flow, neuroinflammation, BBB disruption, brain tissue loss, and long‐term neurological outcomes.

## 2 Materials and Methods

### 2.1 Animals and Drug Administration

C57BL/6 mice (male, 8–10 weeks old, 22–25 g; Jackson Laboratory) were used in the study. All mice were housed in pathogen‐free conditions at the animal facilities under a standardized light–dark cycle and had free access to food and water. All procedures were performed in accordance with the National Institutes of Health Guide for the Care and Use of Laboratory Animals and approved protocols from the Institutional Animal Care and Use Committee (IACUC) of Tulane University.

Colchicine (Selleckchem, S2284) was dissolved in saline to prepare a stock solution (50 mM). Working solutions were freshly prepared before use. A dose‐ranging cohort received colchicine (0.4, 0.8, and 1.6 mg/kg) intraperitoneally (i.p.) at the time of reperfusion. The dosage range was determined based on previous studies [36, 37]. The optimal dose was defined as the minimum effective dose that conferred robust neuroprotection without evidence of hematopoietic toxicity, and 0.8 mg/kg was therefore selected for all subsequent mechanistic and outcome studies.

### 2.2 Transient Middle Cerebral Artery Occlusion (tMCAO)

Transient focal cerebral ischemia was induced by 50‐min occlusion of the left middle cerebral artery (MCA), as we previously described [38, 39]. Briefly, mice were anesthetized with isoflurane, and a silicone‐coated monofilament (Doccol Corporation, 602134PK10Re) was advanced via the left carotid circulation into the internal carotid artery to occlude the MCA for 50 min, followed by filament withdrawal to allow reperfusion. Mice were randomized before surgery. Colchicine or vehicle was administered at reperfusion, and exclusion criteria were applied to all animals based on neurological severity assessed by mNSS 1 h after reperfusion to confirm successful model establishment. Mice with mNSS ≤ 5 were excluded as likely to have incomplete occlusion, whereas those with mNSS > 14 were excluded due to severe neurological impairment or suspected perioperative complications [40]. In total, 13 of 61 mice in the Vehicle group and 10 of 56 mice in the Colchicine group were excluded according to these prespecified criteria. The exclusion rates were similar between treatment groups (21.3% vs. 17.9%). Body temperature was maintained at 36.5°C–37.0°C throughout surgery. Sham‐operated mice underwent identical surgical exposure without filament insertion. After surgery, mice were allowed to recover on a warming pad under close observation for approximately 30 min, until fully awake, and were then returned to their home cages with free access to food and water. Softened food was provided on the cage floor when needed to facilitate postoperative feeding. Animals were monitored daily for general condition and signs of distress. Postoperative analgesia was administered in accordance with the institutional animal care protocol.

### 2.3 Flow Cytometry

At 4 h, 24 h, or 3 days after tMCAO, mice were euthanized under deep isoflurane anesthesia, and peripheral blood, brain, spleen, and femur/bone marrow were collected for flow cytometric analysis, as previously described [41, 42], with additional details provided in Supporting Information.

### 2.4 Assessment of Infarct Volume

At Day 3 after tMCAO, infarct volume was assessed by 2,3,5‐triphenyltetrazolium chloride (TTC) staining as we previously described [43]. Mice were perfused with cold PBS, brains were sectioned into 1‐mm coronal slices, and incubated in 2% TTC (Sigma‐Aldrich, T8877) for 20 min in the dark. Slices were fixed in 4% paraformaldehyde, imaged, and the infarct volume was quantified in ImageJ (National Institutes of Health). Infarct volume was calculated as [(contralateral volume − non_infarcted ipsilateral volume)/contralateral volume] × 100% [43].

### 2.5 Assessment of Neutrophil F‐Actin Polymerization

To quantify neutrophil cytoskeletal remodeling, F‐actin polymerization was assessed by phalloidin staining, as previously described [44]. Briefly, peripheral blood was collected 4 h after tMCAO, and red blood cells were lysed using RBC lysis buffer (BioLegend, 420302). After washing, cells were resuspended and plated onto 0.1% gelatin–coated coverslips (STEMCELL Technologies, 07903) in 24‐well plates for 1 h at 37°C. Cells were then fixed with 4% paraformaldehyde, permeabilized with 0.2% Triton X‐100, and blocked with 3% BSA. Coverslips were incubated with Alexa Fluor 594–conjugated anti‐mouse Ly6G (BioLegend, 127636) and CoraLite Plus 488‐phalloidin (Proteintech, PF00001) for 1 h. After washing, coverslips were mounted with antifade medium containing DAPI (Vector, H‐1500‐10). Images were acquired on a Nikon Eclipse Ti2 confocal microscope (Nikon). F‐actin content was quantified in Ly6G^+^ cells using ImageJ by measuring phalloidin mean fluorescence intensity (MFI) per cell in randomly selected fields; **≥** 20 Ly6G^+^ cells per mouse were analyzed by a blinded investigator, and the mouse‐level average was used for statistical analysis [44].

### 2.6 Immunostaining

Immunostaining was conducted as we previously described [45], with additional staining procedures, antibody information, section‐processing details, and quantification methods provided in Supporting Information. For intravascular neutrophil/NET analysis, brains were collected without transcardial perfusion [46], whereas for fibrin(ogen) deposition and endogenous IgG extravasation, mice were perfused with ice‐cold PBS before tissue collection [42, 45]. Images were acquired using identical settings within each experiment and analyzed in ImageJ using a predefined threshold applied uniformly to all images within the same analysis batch [42]. No additional normalization across samples was applied beyond identical image acquisition settings and uniform analysis parameters within the same experiment.

### 2.7 Cerebral Blood Flow Measurement

To assess post‐reperfusion regional cerebral blood flow (rCBF), two‐dimensional laser speckle contrast imaging (LSCI, RFLSI ZW Laser Speckle Imaging System, RWD Life Science Co.) was performed as previously described [47]. Mice were anesthetized, secured in a stereotaxic frame, and maintained at 37°C during imaging. Following a midline scalp incision, the intact skull was exposed and continuously moistened. Speckle images were acquired at 1 frame/s (interval mode), and 10 consecutive frames were averaged per time point. rCBF was recorded at baseline (pre‐ischemia), during MCA occlusion, and at 4 and 24 h after reperfusion using identical acquisition settings. Regions of interest (ROIs) of identical size were placed over the visible cortical surface of the ipsilateral and contralateral hemispheres on the speckle images. The same ROI size and placement strategy were applied across animals within each experiment. Perfusion was quantified in arbitrary perfusion units and expressed as the ipsilateral‐to‐contralateral ratio (ipsilateral/contralateral × 100%) [47].

### 2.8 Brain Microvessels Preparation

Brain microvessels were prepared as we described previously [48]. Briefly, at 24 h after tMCAO, mice were transcardially perfused with ice‐cold PBS, and the ipsilateral hemispheres were dissected. Cortical tissue was homogenized in ice‐cold PBS using a Dounce homogenizer (Kimble Chase) and centrifuged (1000 × *g*, 5 min, 4°C). The pellet was resuspended in 18% dextran (60–90 kDa; Fisher Scientific) in PBS and centrifuged (1500 × *g*, 20 min, 4°C). The resulting pellet was washed with PBS and passed through a 40‐μm cell strainer; microvessels retained on the strainer were collected and stored at −80°C.

### 2.9 Real‐Time Quantitative PCR Analysis

Real‐time quantitative PCR (RT‐qPCR) was performed as we described previously [42]. Total RNA was isolated from brain microvessels, reverse‐transcribed, and analyzed using TaqMan chemistry. Relative expression was calculated by the $2^{-∆∆C_{t}}$ method with normalization to the indicated housekeeping gene. Probe information is provided in Supporting Information.

### 2.10 Whole‐Blood Phagocytosis Assay

At Day 3 after tMCAO, whole blood was collected into anticoagulant‐coated tubes (EDTA) and kept at room temperature for immediate processing. Phagocytic function was assessed using pHrodo Green *S. aureus* BioParticles conjugate (Invitrogen, P35367) according to the manufacturer's instructions. Briefly, samples were incubated with pHrodo BioParticles at 37°C, then erythrocytes were lysed, leukocytes were surface‐stained with antibodies against CD45 (BioLegend, 103112), Ly6G (BioLegend, 127622), and F4/80 (BioLegend, 123110), and analyzed by flow cytometry. Phagocytosis was quantified as the pHrodo fluorescence MFI within each leukocyte population [49].

### 2.11 Neurological Function Assessments

Neurological deficit assessment was performed by investigators blinded to group assignment as we previously described [50, 51]. During the 14‐day follow‐up, mortality was 3/13 (23.1%) in the tMCAO + Vehicle group and 2/12 (16.7%) in the tMCAO + colchicine group, with no deaths in the sham. Behavioral analyses included only surviving animals at each time point. Sensorimotor function was evaluated on Days 1, 3, 7, and 14 after tMCAO using the modified Neurological Severity Score (mNSS), adhesive removal test, and foot‐fault test. The mNSS (0–18) integrates motor, sensory, reflex, and balance measures. The higher the scores summed, the more severe the deficit is [50]. For the adhesive removal test, mice were trained for 2–3 days before surgery, and latencies to contact and remove an adhesive patch placed on the forepaw were recorded (maximum 120 s) [52]. For the foot‐fault test, mice traversed an elevated wire grid for 2 min, and contralateral forelimb foot‐faults were expressed as a percentage of total steps [50]. Spatial reference memory was assessed on Day 14 using a two‐trial Y‐maze test as previously described [53]. In trial 1, one arm was blocked, and mice explored the two open arms for 10 min; 2 h later (trial 2), all arms were opened and mice explored for 5 min. Arm entries and time spent in each arm were tracked and analyzed using ANY‐maze software (Stoelting). Spatial reference memory was quantified as the percentage of time spent in the novel arm during trial 2 [53].

### 2.12 Quantitation of Brain Tissue Loss

To quantify brain tissue loss at 14 days after tMCAO, six evenly spaced coronal brain slices (8 μm thick) from bregma: +1.54 to −2.55 mm, with 700 μm interval, were stained with antibodies Anti‐MAP2 mouse Ab (Sigma, M4403) as we described previously [48]. Images were captured using identical camera settings with a Zeiss Axio ScanZ. 1 Slide Scanner (Zeiss) and analyzed using ImageJ. The volume of each hemisphere was calculated by multiplying the area of tissue loss by the distances between the analyzed brain sections. Tissue loss was expressed as (V_contra − (V_ipsi_remaining))/V_contra × 100% [48].

### 2.13 Neutrophil Depletion

Neutrophils were depleted with Purified anti‐mouse Ly‐6G Antibody (BioLegend, 127650) as previously described [54]. Mice were injected intraperitoneally with 10 mg/kg/day Purified anti‐mouse Ly‐6G Antibody or isotype control (BioLegend, 400566) 2 days before and 1 day after tMCAO. Depletion efficacy was confirmed in peripheral blood by flow cytometry before downstream analyses.

### 2.14 In Vitro Assays

Detailed procedures for human brain microvascular endothelial cells (HBMVEC) culture and hypoxia/reoxygenation (H/R) treatment, FITC–dextran trans‐endothelial permeability, MTT viability assay, and RT–qPCR are provided in Supporting Information [42, 55, 56, 57, 58, 59, 60].

### 2.15 Statistical Analysis

This study was designed as a hypothesis‐generating preclinical investigation. Sample sizes were determined based on pilot experiments, our prior experience with the tMCAO model, and previous studies using similar outcome measures [45, 48, 51, 61, 62]. Investigators were blinded to group allocation throughout treatment administration, data acquisition, and endpoint analyses. Statistical analyses were performed using Prism 10 (GraphPad Software). Data are presented as mean ± SEM. Normality was assessed with the Shapiro–Wilk test and homogeneity of variances with Levene's test. Comparisons between two groups were performed using an unpaired two‐tailed *t* test, whereas comparisons among three or more groups were analyzed by one‐way or two‐way ANOVA, as appropriate, followed by Tukey's multiple‐comparisons test. For longitudinal behavioral data repeatedly measured in the same animals, including mNSS, adhesive removal, and foot‐fault tests, two‐way repeated‐measures ANOVA with Geisser–Greenhouse correction was used, followed by Tukey's multiple‐comparisons test. When parametric assumptions were not satisfied, nonparametric tests (e.g., Mann–Whitney *U* test) were used. All tests were two‐sided, and *p* < 0.05 was considered statistically significant. Because of the exploratory nature of the study, no formal study‐wide adjustment for multiple comparisons was applied.

## 3 Results

### 3.1 Dose‐Range Effects of Colchicine on Peripheral Leukocytes and Brain Infarction in tMCAO Mice

**FIGURE 1:**
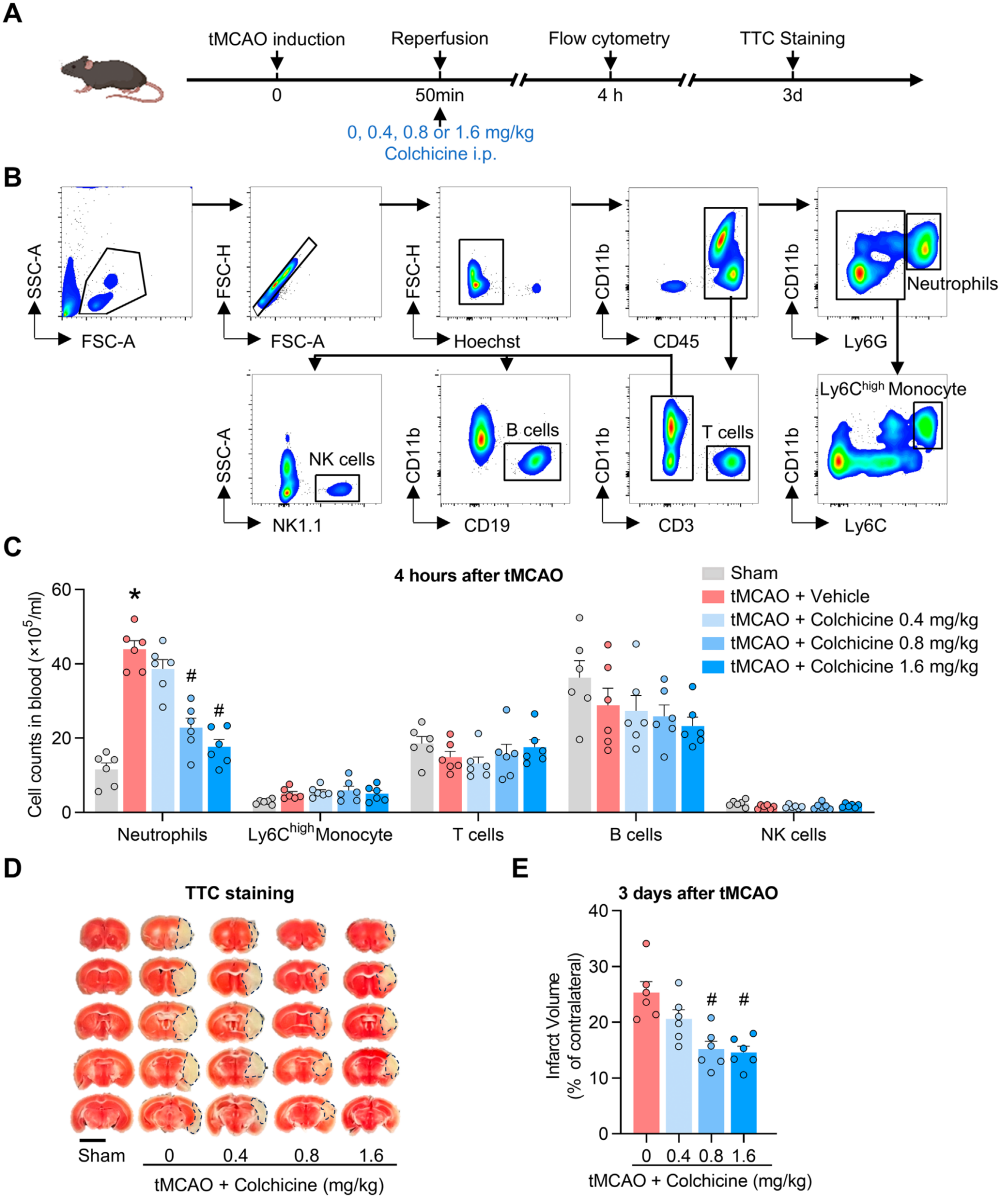
Dose‐range effects of colchicine on acute brain injury after tMCAO in mice. Mice underwent 50‐min tMCAO and received colchicine (0.4, 0.8, or 1.6 mg/kg) or vehicle at reperfusion. Peripheral blood was collected at 4 h after tMCAO, and brains were harvested at Day 3 after tMCAO. (A) Schematic diagram of the experimental design. (B) Flow cytometric gating strategy for circulating neutrophils in mice (CD45^+^ CD11b^+^ Ly6G^+^), Ly6C^high^ monocytes (CD45^+^ CD11b^+^ Ly6G^−^ Ly6C^high^), T cells (CD45^+^ CD11b^−^ CD3^+^), B cells (CD45^+^ CD11b^−^ CD3^−^ CD19^+^), and NK cells (CD45^+^ CD3^−^ NK1.1^+^). (C) Cell counts of leukocyte subtypes at 4 h after tMCAO in peripheral blood. (D, E) Representative TTC staining images (D) and quantification (E) of infarct volume at Day 3 after tMCAO. Scale bar: 5 mm. Black dashed outline indicating the infarct region. *n* = 6 mice per group. Mean ± SEM; *p < 0.05 vs. Sham; ^#^ *p* < 0.05 vs. tMCAO + Vehicle. One‐way ANOVA.

Using a mouse tMCAO model (50‐min MCA occlusion followed by reperfusion), we first assessed how dosing colchicine at reperfusion affected peripheral leukocytes at 4 h by flow cytometry. The experimental timeline and treatment regimen are illustrated in the flow chart (Figure 1A). The gating strategy is shown in Figure 1B. Compared with the sham group, tMCAO induced a significant increase in the number of circulating neutrophils. Colchicine treatment reduced neutrophil counts, with greater suppression at doses ≥ 0.8 mg/kg at 4 h after tMCAO (0.4 mg/kg: 12.2% reduction, *p* = 0.47; 0.8 mg/kg: 48.1% reduction, *p* < 0.0001; 1.6 mg/kg: 59.8% reduction, *p* < 0.0001; vs. tMCAO + Vehicle, respectively; Figure 1C). In addition, colchicine treatment did not significantly alter the numbers of Ly6C^high^ monocytes, T cells, B cells, and NK cells compared with the tMCAO + Vehicle group (Figure 1C). We next assessed dose‐range effects of colchicine in ischemic brain infarction at 3 days after tMCAO. Although 0.4 mg/kg reduced circulating neutrophil counts at 4 h, it did not translate into a significant reduction of infarct volume at Day 3 (0.4 mg/kg: 18.5% reduction, *p* = 0.1926; vs. tMCAO + Vehicle; Figure 1D,E). In contrast, both 0.8 and 1.6 mg/kg significantly decreased infarct size with no difference between the two doses (0.8 mg/kg: 40.0% reduction, *p* = 0.0011; 1.6 mg/kg: 42.3% reduction, *p* = 0.006; vs. tMCAO + Vehicle, respectively; Figure 1D,E). Taken together, these results indicated that dose‐dependent neuroprotection by colchicine is associated with the suppression of peripheral neutrophil accumulation, and identified 0.8 mg/kg as the optimal (minimum effective) dose for investigations of therapeutic effects and underlying pathological mechanisms.

### 3.2 Colchicine Inhibits tMCAO‐Induced Circulating Neutrophil Increase and Activation in Mice

**FIGURE 2:**
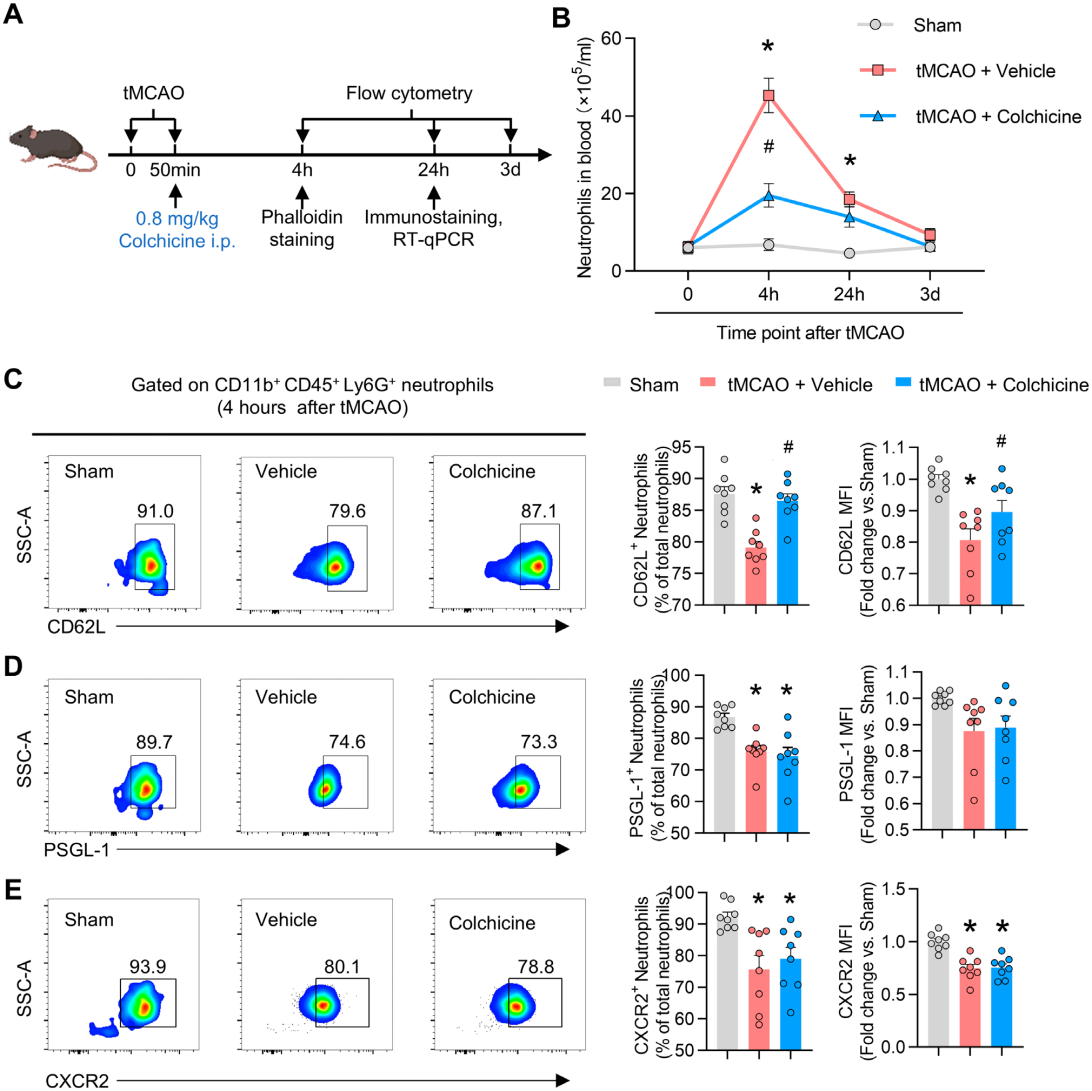
Colchicine attenuates tMCAO‐induced circulating neutrophil mobilization and activation. Mice were subjected to 50‐min tMCAO and received colchicine (0.8 mg/kg) or vehicle at reperfusion. Peripheral blood was collected from Sham, tMCAO + Vehicle, and tMCAO + Colchicine groups at 4 h, 24 h, and 3 days after tMCAO. (A) Schematic diagram of the experimental design. (B) Counts of circulating neutrophils at 4 h, 24 h, and 3 days after tMCAO. *n* = 6–8 mice per group. (C–E) Flow cytometry analysis of neutrophil activation and adhesion markers: CD62L (C), PSGL‐1 (D), and CXCR2 (E) at 4 h after tMCAO. Left: Gating strategy; middle: Percentage; right: Mean fluorescence intensity (MFI). *n* = 8 mice per group. Mean ± SEM; *p < 0.05 vs. Sham; ^#^ *p* < 0.05 vs. tMCAO + Vehicle. One‐way ANOVA.

Next, we examined the effects of colchicine on circulating neutrophil dynamics and phenotype after tMCAO. Mice underwent 50‐min tMCAO and received colchicine (0.8 mg/kg, i.p.) at reperfusion. Peripheral blood was collected at 4 h, 24 h, and 3 days after tMCAO for flow cytometry analysis (Figure 2A; Figure S1). The results showed that tMCAO induced a rapid neutrophil surge peaking at 4 h and remaining elevated at 24 h, which was blunted by colchicine (56.9% reduction at 4 h, *p* = 0.0001; 24.6% reduction at 24 h, *p* = 0.2486; vs. tMCAO + Vehicle; Figure 2B); by Day 3, neutrophil counts converged across groups (Figure 2B).

To investigate the effect of colchicine on neutrophil activation after tMCAO in mice, we further analyzed activation and adhesion markers of circulating neutrophils at 4 h after tMCAO. Consistent with an activated phenotype, tMCAO significantly reduced the expression of CD62L, PSGL‐1, and CXCR2 compared with sham. Colchicine significantly attenuated the tMCAO‐induced downregulation of CD62L on neutrophils (Figure 2C), but did not significantly affect CXCR2 or PSGL‐1 expression at 4 h after tMCAO (Figure 2D,E). Together, these findings indicate that colchicine limits tMCAO‐induced acute systemic neutrophil response and partially restrains early neutrophil activation, at least in part by preserving CD62L expression.

### 3.3 Colchicine Suppresses Neutrophil F‐Actin Polymerization in Peripheral Blood After tMCAO in Mice

**FIGURE 3:**
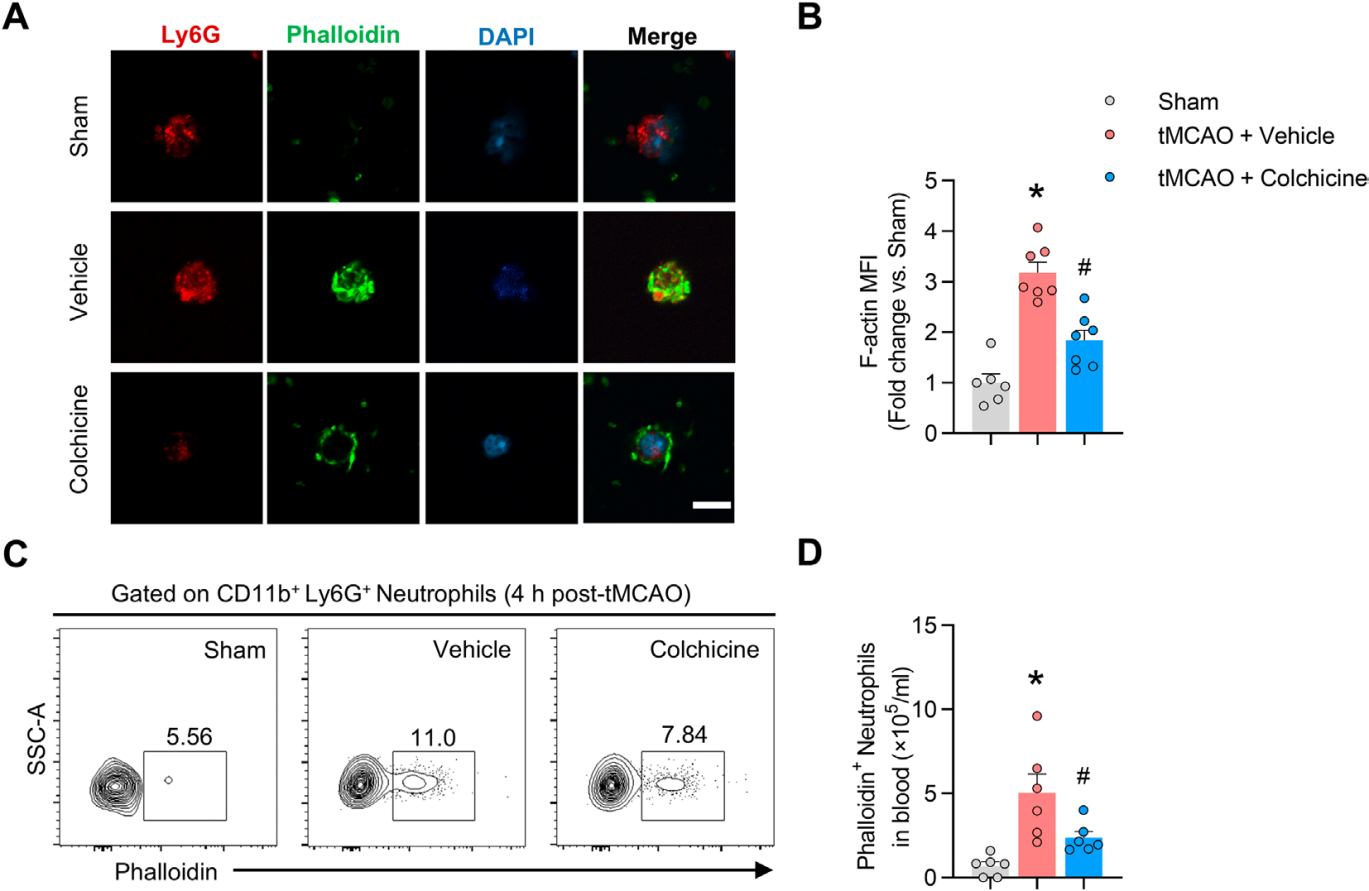
Colchicine suppresses neutrophil F‐Actin polymerization in peripheral blood after tMCAO. Mice were subjected to 50‐min tMCAO and received colchicine (0.8 mg/kg) or vehicle at reperfusion. Peripheral blood was collected from Sham, tMCAO + Vehicle, and tMCAO + Colchicine groups at 4 h after tMCAO induction. (A, B) Representative images (A) and quantification (B) of F‐Actin polymerization in neutrophils. Scale bar: 10 μm. *N* = 6–7 mice per group. (C, D) Flow cytometric gating strategy (C) and quantification (D) of phalloidin^+^ neutrophils. *n* = 6 mice per group. Mean ± SEM; *p < 0.05 vs. Sham; ^#^ *p* < 0.05 vs. tMCAO + Vehicle. One‐way ANOVA.

To validate the effects of Colchicine on F‐actin polymerization in neutrophils, we collected peripheral blood at 4 h after tMCAO, and found a marked increase in F‐actin polymerization in circulating neutrophils after tMCAO by phalloidin staining (232.3% increase vs. Sham, *p* = 0.0003), which was significantly reduced by colchicine administration (33.3% reduction vs. tMCAO + Vehicle, *p* = 0.0343; Figure 3A,B). Consistently, flow cytometry analysis revealed a reduced phalloidin^+^ neutrophil population in the peripheral blood of colchicine‐treated mice compared with the vehicle group (52.8% reduction vs. tMCAO + Vehicle, *p* = 0.0426; Figure 3C,D; Figure S2). These results suggest that tubulin disruption by colchicine inhibits actin remodeling in neutrophils, thereby limiting early inflammatory activation and effector function.

### 3.4 Colchicine Reduces NET Formation and Improves Regional Cerebral Blood Flow After tMCAO in Mice

**FIGURE 4:**
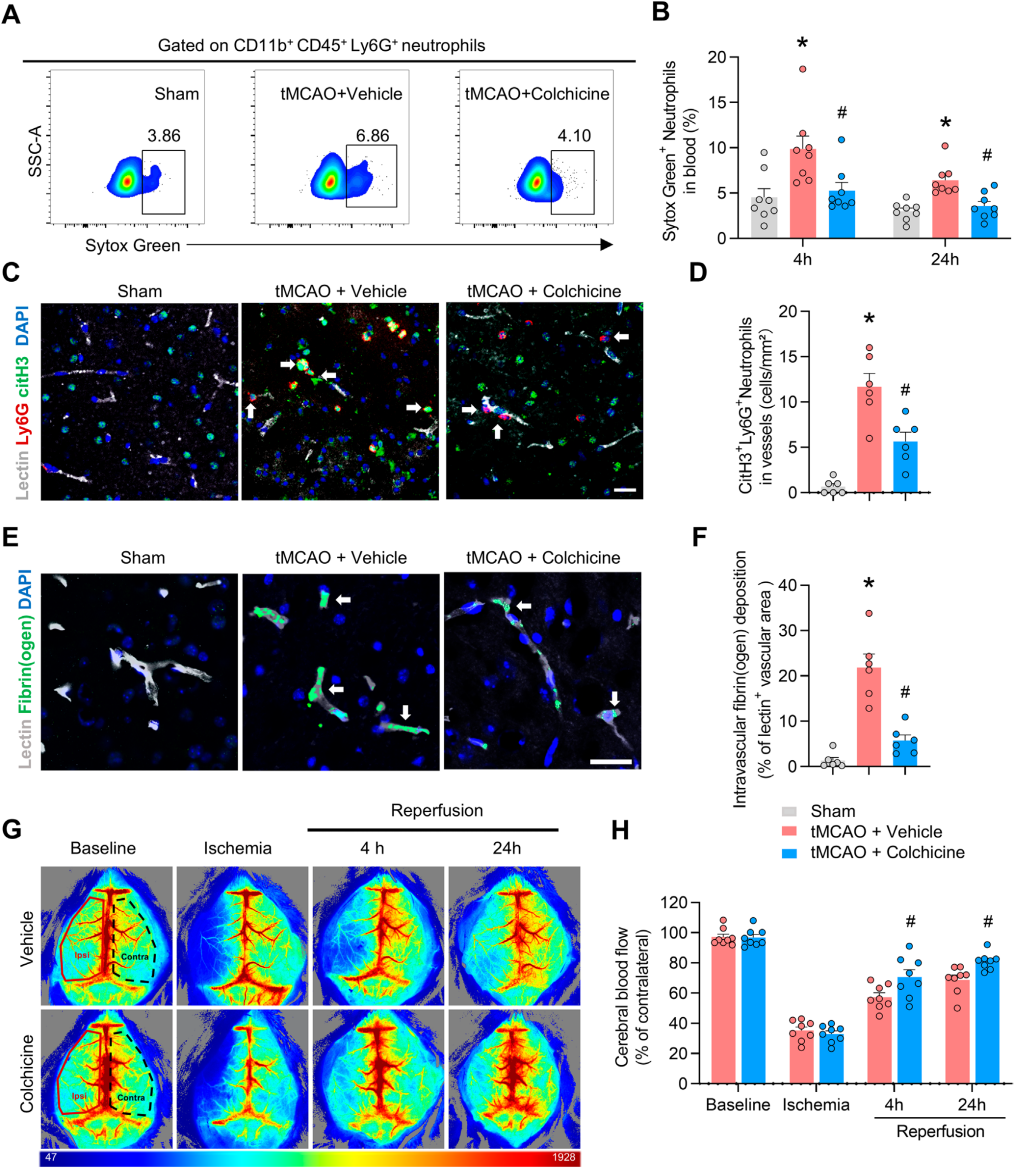
Colchicine reduces NET formation and microvascular thrombosis and improves cerebral blood flow after tMCAO. Mice were subjected to 50‐min tMCAO and received colchicine (0.8 mg/kg) or vehicle at reperfusion. Peripheral blood and brains were collected from Sham, tMCAO + Vehicle, and tMCAO + Colchicine groups at 24 h after tMCAO. (A, B) Flow cytometric gating strategy (A) and quantification (B) of SYTOX Green^+^ neutrophils in peripheral blood at 4 and 24 h after tMCAO. *n* = 8 mice per group. (C, D) Representative images (C) and quantification (D) of CitH3^+^ Ly6G^+^ neutrophils in lectin^+^ cerebral microvessels (white arrows). Scale bar: 25 μm. *n* = 6 mice per group. (E, F) Representative images (E) and quantification (F) of fibrin (ogen) deposition within lectin^+^ cerebral microvessels (white arrows). Scale bar: 25 μm. *n* = 6 mice per group. (G) Representative laser speckle contrast imaging perfusion maps of CBF at the indicated time points. Representative regions of ROIs used for regional CBF quantification are shown in the baseline images. Ipsi, ipsilateral ROI (red solid outline); Contra, contralateral ROI (black dashed outline). (H) Quantification of regional CBF, expressed as the ipsilateral‐to‐contralateral perfusion ratio (%). *n* = 8 mice per group. Mean ± SEM; *p < 0.05 vs. Sham, and ^#^ *p* < 0.05 vs. tMCAO + Vehicle. One‐way ANOVA in (B, D, and F), unpaired two‐tailed *t* test in (H).

To further examine the effects of colchicine on neutrophil‐derived inflammatory responses after tMCAO, we assessed intravascular NET formation and intravascular fibrin(ogen) deposition. Our results showed that circulating neutrophils exhibited a marked increase in extracellular DNA release at 4 h after tMCAO (117.9% increase vs. Sham), and sustained the elevation at 24 h (114.6% increase vs. Sham), which was significantly reduced by colchicine treatment at both time points (46.5% reduction at 4 h, *p* = 0.0227; 44.6% reduction at 24 h, *p* = 0.0015; vs. tMCAO + Vehicle; Figure 4A,B; Figure S3). In parallel, immunofluorescence co‐staining with lectin showed that tMCAO increased the presence of CitH3^+^ Ly6G^+^ neutrophils inside the peri‐infarct cerebral microvasculature, which was significantly reduced by colchicine treatment (54.5% reduction vs. tMCAO + Vehicle, *p* = 0.0030; Figure 4C,D). These results indicate that colchicine treatment effectively inhibits tMCAO‐induced intravascular NET formation. We next assessed intravascular fibrin(ogen) deposition and cerebral perfusion after tMCAO. Our data showed that intravascular fibrin(ogen) deposition in peri‐infarct regions was elevated at 24 h after tMCAO and was significantly reduced by colchicine administration compared with the vehicle group (73.6% reduction vs. tMCAO + Vehicle, *p* < 0.0001; Figure 4E,F). Consistent with these vascular changes, colchicine treatment also significantly improved regional cerebral blood flow compared with the vehicle group after tMCAO (23.1% increase at 4 h, *p* = 0.0323; 17.8% increase at 24 h, *p* = 0.0057; vs. tMCAO + Vehicle; Figure 4G,H). Together, these results indicate that colchicine attenuates NET‐associated thrombo‐inflammatory vascular changes after tMCAO and is accompanied by improved regional cerebral perfusion.

### 3.5 Colchicine Treatment Attenuates tMCAO‐Induced Cerebral Vascular Pro‐Inflammatory Activation and BBB Disruption in Mice

**FIGURE 5:**
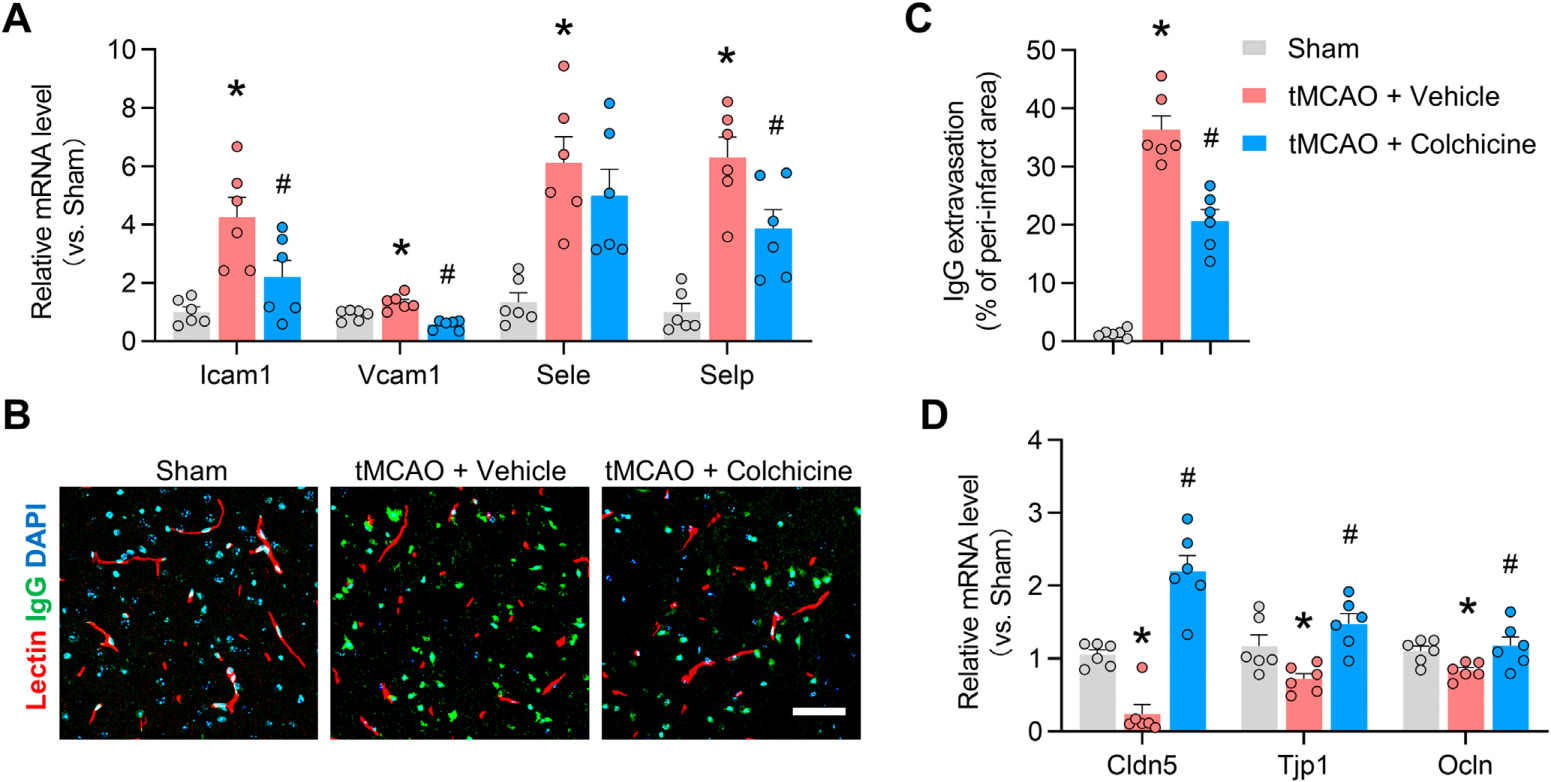
Colchicine treatment attenuates tMCAO‐induced cerebral vascular pro‐inflammatory activation and BBB disruption in mice. Mice were subjected to 50‐min tMCAO and received colchicine (0.8 mg/kg) or vehicle at reperfusion. Brains were harvested at 24 h after tMCAO from Sham, tMCAO + Vehicle, and tMCAO + Colchicine groups. (A) RT–qPCR analysis of Icam1, Vcam1, Sele, and Selp mRNA levels in isolated cerebral microvessels. (B, C) Representative images (B) and quantitation (C) of IgG extravasation. Scale bar: 50 μm. *n* = 6 mice per group. (D) RT–qPCR analysis of Cldn5, Ocln, and Tjp1 mRNA levels in isolated cerebral microvessels. *n* = 4 mice per group. Mean ± SEM; *p < 0.05 vs. Sham; ^#^ *p* < 0.05 vs. tMCAO + Vehicle. One‐way ANOVA.

At 24 h after tMCAO, cerebrovascular expression of adhesion molecules Icam1, Vcam1, and Selp was significantly increased, and colchicine markedly reduced these elevations compared with vehicle (Icam1: 48.2%, *p* = 0.0384; Vcam1: 57.4%, *p* < 0.0001; Sele: 18.4%, *p* = 0.5542; Selp: 39.0%, *p* = 0.0227 vs. tMCAO + Vehicle, respectively; Figure 5A). Consistently, BBB permeability was substantially increased in vehicle‐treated mice, whereas IgG extravasation was significantly reduced by colchicine (50.6% reduction vs. tMCAO + Vehicle, *p* < 0.0001; Figure 5B,C), accompanied by partial restoration of tight‐junction transcripts (Figure 5D). These results show that colchicine treatment can attenuate cerebral vascular pro‐inflammation and reduce BBB disruption after tMCAO in mice.

To determine whether colchicine has direct cerebrovascular protective effects, we tested colchicine in an in vitro H/R + IL‐1β BBB injury model (Figure S4A). Although H/R + IL‐1β increased endothelial permeability (H/R + IL‐1β + Vehicle: 54.1% increase, *p* = 0.0215; H/R + IL‐1β + Colchicine: 63.2% increase, *p* = 0.0092 vs. Control), colchicine did not reduce FITC–dextran flux (*p* = 0.92, H/R + IL‐1β + Colchicine vs. H/R + IL‐1β + Vehicle; Figure S4B), indicating colchicine had no direct effect on trans‐endothelial permeability under hypoxia/inflammation conditions. Notably, colchicine did not affect cell viability across treatment groups and did not restore the H/R + IL‐1β–induced decrease in TJP1 expression (Figure S4C,D). In contrast, colchicine significantly attenuated H/R + IL‐1β–induced ICAM1 and VCAM1 upregulation (Figure S4E). These data suggest the observed BBB protection by colchicine in the tMCAO mouse model is likely cerebrovascular indirect effects.

### 3.6 Colchicine Attenuates Leukocyte Infiltration and Neutrophil Pro‐Inflammatory Activation in the Brain After tMCAO in Mice

**FIGURE 6:**
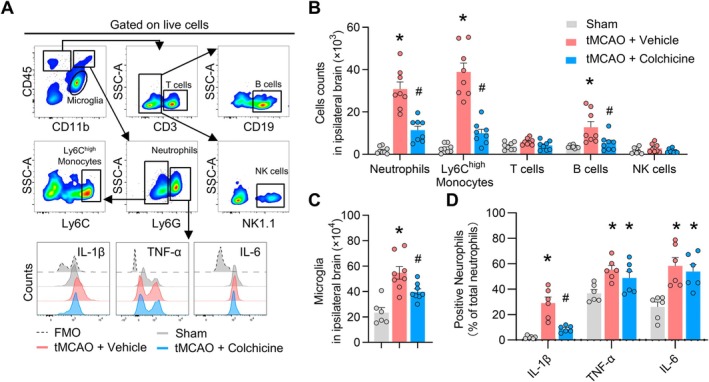
Colchicine attenuates brain leukocyte infiltration and neutrophil pro‐inflammatory activation after tMCAO in mice. Mice were subjected to 50‐min tMCAO and received colchicine (0.8 mg/kg) or vehicle at reperfusion. Brains were harvested at 24 h after tMCAO from Sham, tMCAO + Vehicle, and tMCAO + Colchicine groups. (A) Flow cytometric gating strategy for brain immune cell subset: Neutrophils (CD11b^+^ CD45^high^ Ly6G^+^), Ly6C^high^ Monocytes (CD11b^+^ CD45^high^ Ly6G^−^ Ly6C^high^), T cells (CD11b^−^ CD45^high^ CD3^+^), B cells (CD11b^−^ CD45^high^ CD19^+^), NK cells (CD45^high^ CD3^−^ NK1.1^+^), and microglia (CD45^int^ CD11b^+^). (B, C) Counts of brain‐infiltrating leukocyte subsets (B) and microglia (C). *n* = 8 mice per group. (D) Expression of IL‐1β, IL‐6, and TNF‐α in brain‐infiltrating neutrophils. FMO, fluorescence minus one. *n* = 6 mice per group. Mean ± SEM; *p < 0.05 vs. Sham; ^#^ *p* < 0.05 vs. tMCAO + Vehicle. One‐way ANOVA.

Next, we asked what the effects of colchicine were on brain leukocyte infiltration and neutrophil pro‐inflammatory activation at 24 h after tMCAO in mice. The gating strategy of flow cytometry analysis for immune cell subsets is shown in Figure 6A and Figure S5. The results showed that colchicine treatment significantly reduced brain infiltration of neutrophils, Ly6C^high^ monocytes, and B cells, as well as the number of microglia, in tMCAO brains compared with the vehicle group (neutrophils: 63.1%, *p* < 0.0001; Ly6C^high^ monocytes: 74.4%, *p* < 0.0001; B cells: 58.1%, *p* = 0.0186; microglia: 27.3%, *p* = 0.0288; vs. tMCAO + Vehicle, respectively; Figure 6B,C). In contrast, colchicine did not affect the number of T cells and NK cells in the brain of tMCAO mice (Figure 6B,C). In addition, colchicine treatment significantly decreased the production of pro‐inflammatory cytokine IL‐1β in brain‐infiltrating neutrophils at 24 h after tMCAO (IL‐1β: 70.2%, *p* = 0.0004; TNFα: 11.9%, *p* = 0.4722; IL‐6: 7.4%, *p* = 0.8433 vs. tMCAO + Vehicle, respectively; Figure 6D), suggesting colchicine attenuates neuroinflammation by reducing leukocyte brain infiltration, microglial accumulation, and neutrophil pro‐inflammatory activation in the brain after tMCAO in mice.

### 3.7 Colchicine Does Not Exacerbate Post‐Stroke Immunosuppression in Mice

**FIGURE 7:**
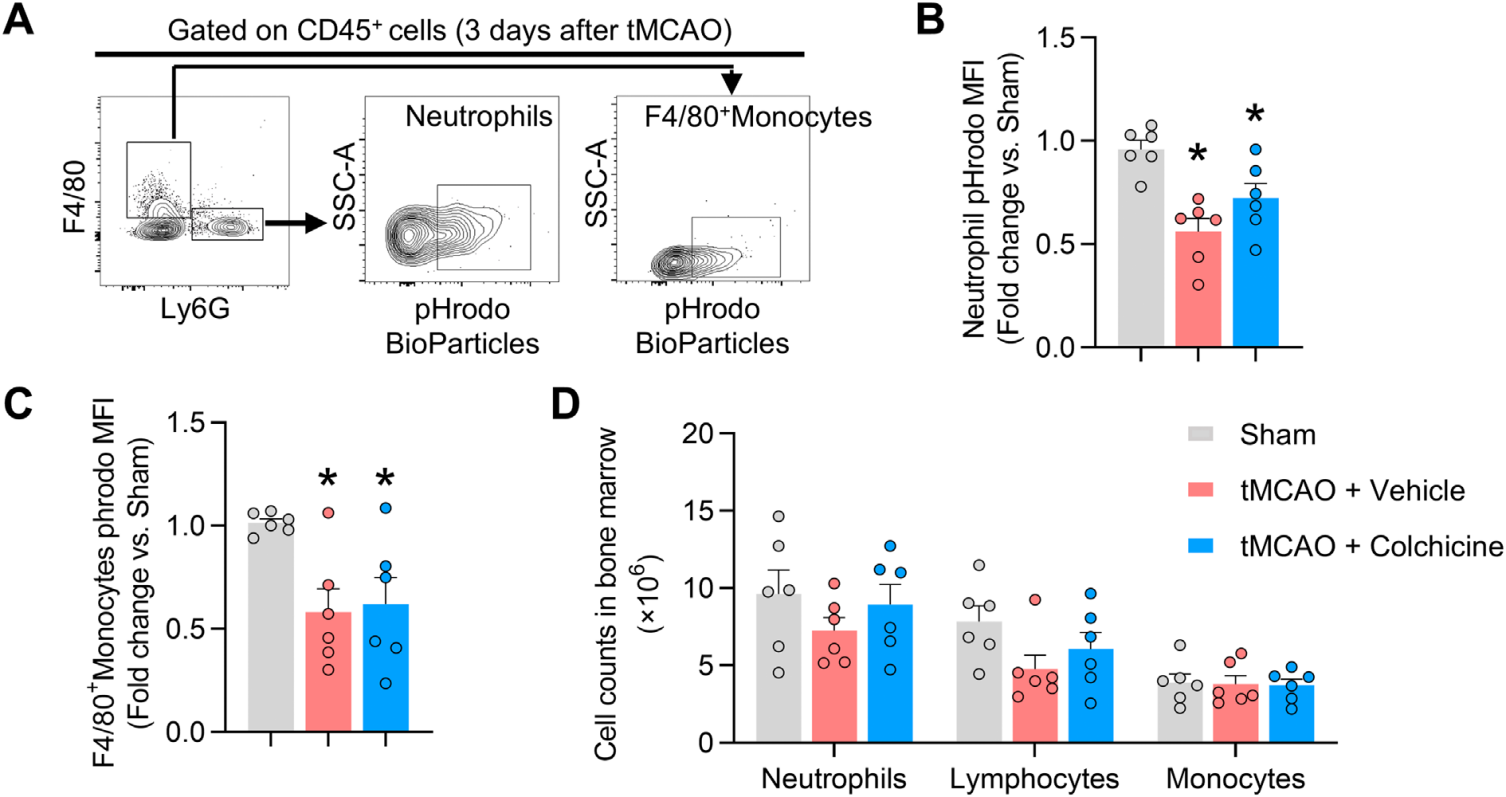
Colchicine does not exacerbate post‐stroke immunosuppression in mice. Mice were subjected to 50‐min tMCAO and received colchicine (0.8 mg/kg) or vehicle at reperfusion. Peripheral blood and bone marrow were collected from Sham, tMCAO + Vehicle, and tMCAO + Colchicine groups at Day 3 after tMCAO. (A) Flow cytometric gating strategy for circulating neutrophils and F4/80^+^ monocytes in the pHrodo BioParticles phagocytosis assay. (B, C) Quantification of pHrodo BioParticles immunofluorescence intensity in circulating neutrophils (B) and F4/80^+^ monocytes (C). *n* = 6 mice per group. (D) Counts of neutrophils, lymphocytes, and monocytes in bone marrow. *n* = 6 mice per group. Mean ± SEM; *p < 0.05 vs. Sham. One‐way ANOVA.

Given the concern that anti‐inflammatory interventions might aggravate post‐stroke immunosuppression [63, 64], we evaluated whether colchicine (0.8 mg/kg) alters innate immune function. At Day 3 after tMCAO, whole‐blood phagocytosis was assayed by pHrodo BioParticles (Figure 7A; Figure S6). Compared with sham, phagocytic MFI was reduced in circulating neutrophils (41.7% reduction vs. Sham, *p* = 0.0008) and F4/80^+^ monocytes (42.5% reduction vs. Sham, *p* = 0.0198) at 3 days after tMCAO, but colchicine treatment at reperfusion did not further depress phagocytosis (neutrophils: *p* = 0.1712; F4/80^+^ monocytes: *p* = 0.9598; tMCAO + Colchicine vs. tMCAO + Vehicle; Figure 7B,C). Moreover, colchicine did not deplete hematopoietic reserves, as bone‐marrow cellularity and neutrophil counts were unchanged compared with vehicle (Figure 7D). These data indicate that colchicine at the effective neuroprotective dose does not further alter post‐stroke innate immune function or deplete myeloid reservoirs at Day 3 after tMCAO in mice, supporting its safety as an adjunctive therapeutic strategy.

### 3.8 Colchicine Improves Long‐Term Neurological Outcomes and Reduces Brain Tissue Loss After tMCAO in Mice

**FIGURE 8:**
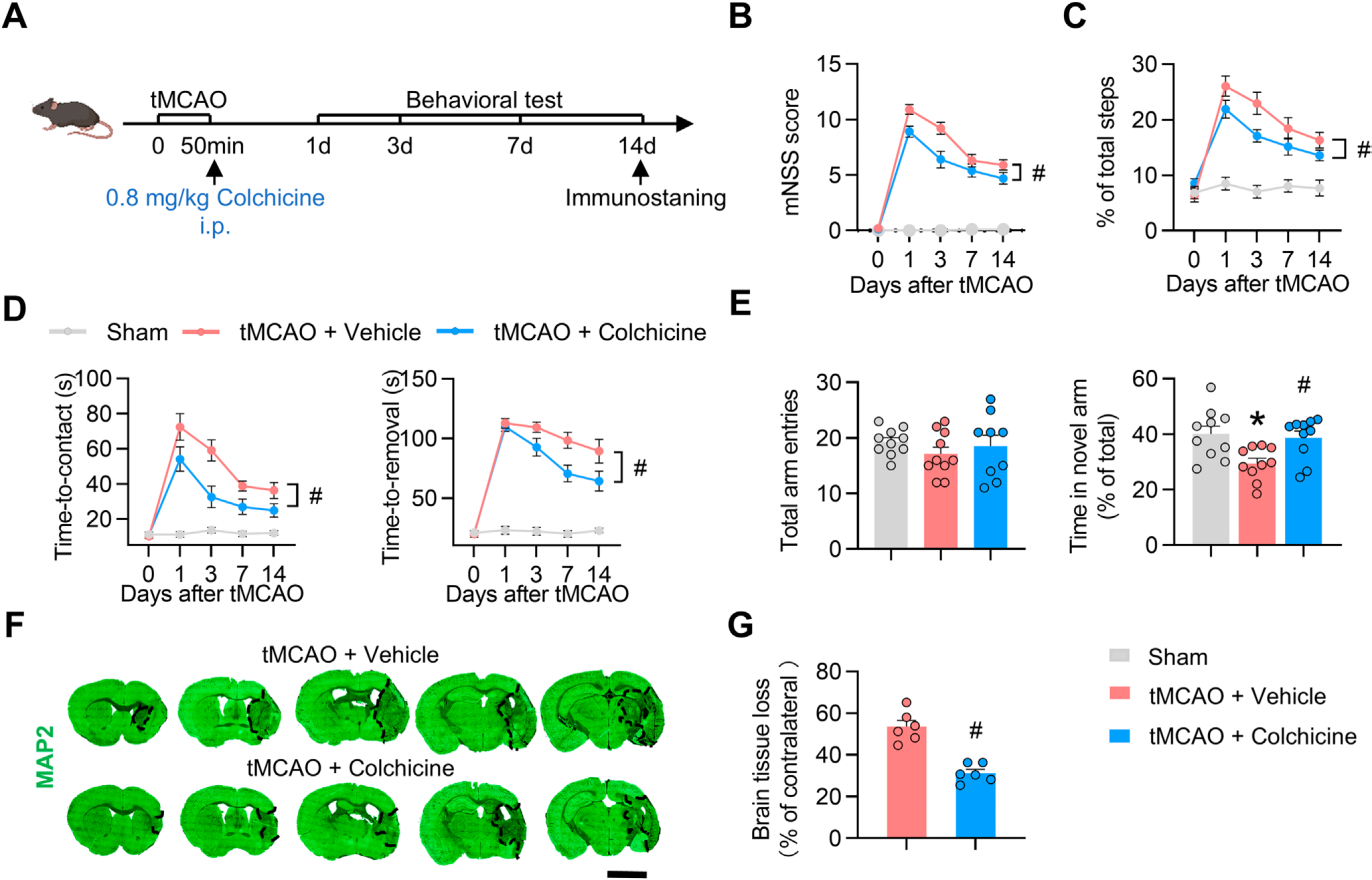
Colchicine improves long‐term neurological outcomes and reduces brain tissue loss after tMCAO in mice. Mice were subjected to 50‐min tMCAO and received colchicine (0.8 mg/kg) or vehicle at reperfusion. Neurobehavioral assessments were performed up to Day 14, and brains were harvested from Sham, tMCAO + Vehicle, and tMCAO + Colchicine groups at Day 14 after tMCAO. (A) Schematic diagram of the experimental design. (B–D) mNSS (B), foot fault test (C), and adhesive removal test including time to contact and time to remove (D). *n* = 10 mice per group. (E) Y‐maze, two‐trial spatial reference memory test at Day 14 after tMCAO. Left: Total arm entries; right: Percentage of time spent in the novel arm, *n* = 10 mice per group. (F, G) Representative MAP2 staining images (F) and quantification (G) of brain tissue loss at Day 14 after tMCAO. Black dashed outline indicating the boundary of tissue loss. Scale bar: 5 mm. *n* = 6 mice per group. Mean ± SEM; *p < 0.05 vs. Sham; ^#^ *p* < 0.05 vs. tMCAO + Vehicle. Two‐way repeated‐measures ANOVA in (B–D), one‐way ANOVA in (E), and unpaired two‐tailed *t* test in (G).

Next, we assessed a series of neurobehavioral tests to evaluate the effects of colchicine treatment on neurological deficits up to 14 days after tMCAO (Figure 8A). We found that colchicine‐treated mice significantly improved performance in the mNss score, foot‐fault test, and adhesive removal test for sensorimotor function (Figure 8B–D). In the Y‐maze two‐trial spatial reference memory test, total arm entries did not differ significantly between groups; however, colchicine treatment increased the percentage of time spent in the novel arm compared with vehicle‐treated mice (31.2% increase vs. tMCAO + Vehicle, *p* = 0.0300; Figure 8E), suggesting improved spatial reference memory at Day 14 after tMCAO. Moreover, brain tissue loss was quantified at 14 days after tMCAO via MAP2 staining. Our results showed colchicine treatment reduced tMCAO‐induced brain tissue loss at 14 days (27.5% reduction vs. tMCAO + Vehicle, *p* < 0.0001; Figure 8F,G). Taken together, these results suggest that colchicine treatment at the reperfusion after tMCAO may reduce brain tissue damage and promote long‐term recovery after tMCAO.

### 3.9 The Neuroprotective Effect of Colchicine Following tMCAO in Mice Is Neutrophil‐Dependent

**FIGURE 9:**
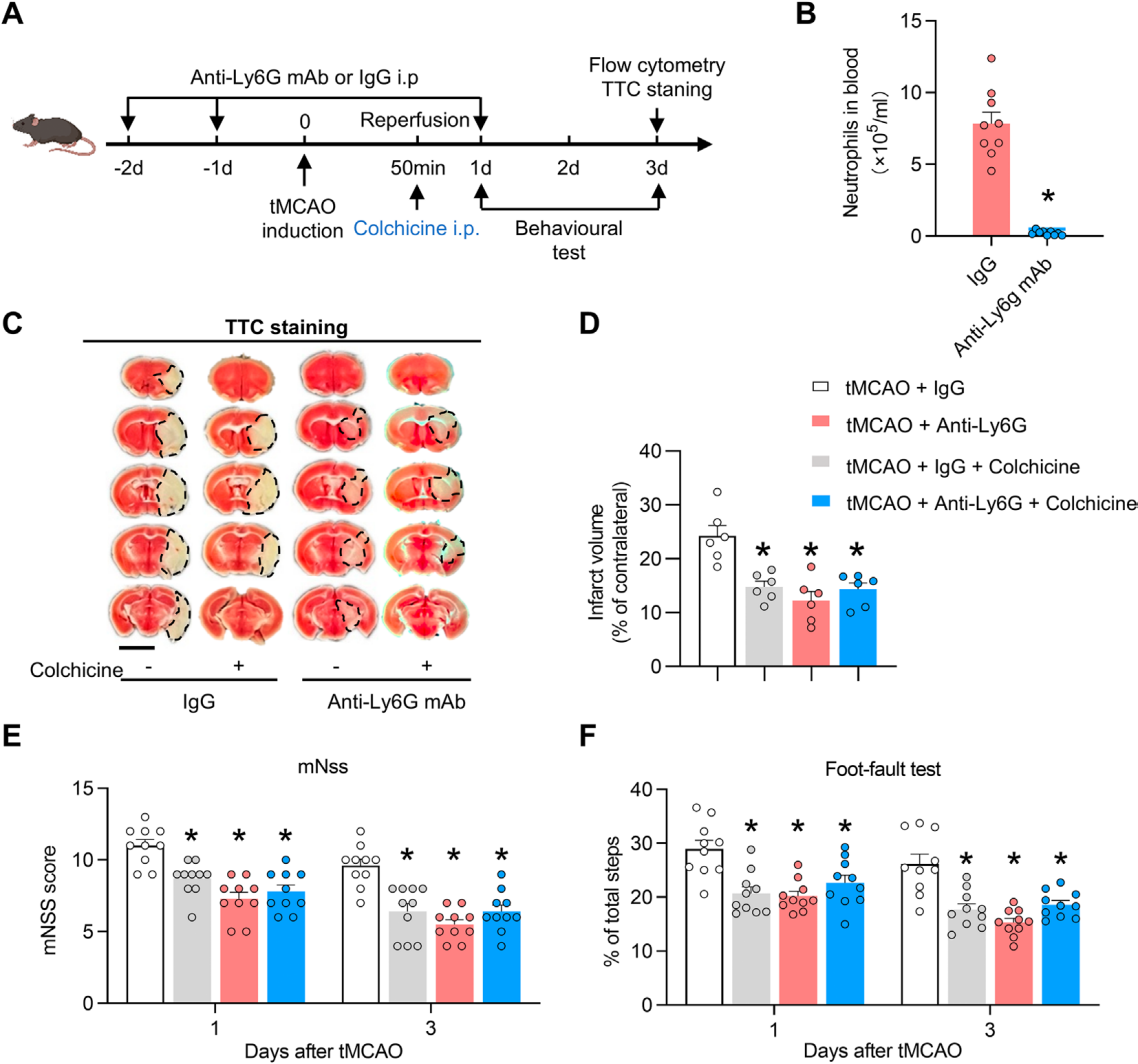
The neuroprotective effect of colchicine is neutrophil‐dependent after tMCAO in mice. Mice were subjected to 50‐min tMCAO and were administered anti‐Ly6G mAb or isotype IgG (10 mg/kg/day, i.p.) at 2 days before and 1 day after tMCAO to deplete neutrophils. Colchicine (0.8 mg/kg) or vehicle was given at reperfusion. Brains were harvested from tMCAO + IgG, tMCAO + Anti‐Ly6G, tMCAO + IgG + Colchicine, and tMCAO + Anti‐Ly6G + Colchicine groups at Day 3 after tMCAO. (A) Schematic diagram of the experimental design. (B) Quantification of circulating neutrophils in the mice receiving anti‐Ly6G mAb or IgG. *n* = 9 mice per group. Mean ± SEM; *p < 0.05 vs. IgG. (C, D) Representative TTC staining images (C) and quantification (D) of brain infarct volume at Day 3 after tMCAO. Scale bar: 5 mm. Black dashed outline indicating the infarct region. *n* = 6 mice per group. (E, F) mNSS (E) and foot fault test (F) at Day 1 and 3 after tMCAO. *n* = 10 mice per group. Mean ± SEM; *p < 0.05 vs. tMCAO + IgG. unpaired two‐tailed *t* test in (B), one‐way ANOVA in (D–F).

To determine whether colchicine has neutrophil‐independent pharmacological actions after tMCAO, a neutrophil depletion approach was applied in mice by receiving anti‐Ly6G mAb or IgG before and after tMCAO induction (Figure 9A). Flow cytometry confirmed that anti‐Ly6G mAb administration eliminated > 90% of neutrophils in peripheral blood (Figure 9B). In tMCAO mice receiving anti‐Ly6G mAb, a reduction of ischemic brain infarction was observed; however, colchicine provided no additional reduction in the infarct size compared to neutrophil depletion alone (*p* = 0.7822; tMCAO + Anti‐Ly6G vs. tMCAO + Anti‐Ly6G + Colchicine; Figure 9C,D). Consistent with brain infarct reduction, colchicine treatment showed no additive improvement on neurological deficits compared to neutrophil depletion after tMCAO in mice (Figure 9E,F). These results indicate that the neuroprotective effects of colchicine in the tMCAO mouse model are mainly neutrophil‐dependent.

## 4 Discussion

In the present study, we tested colchicine as a potent modulator of post‐reperfusion thrombo‐inflammation in tMCAO mice. Colchicine administered at reperfusion selectively restrained early systemic neutrophil responses, cytoskeletal remodeling, activation, and NET formation, leading to reduced fibrin(ogen)‐associated intravascular deposition and improved regional cerebral perfusion. These upstream effects were associated with attenuated cerebrovascular inflammatory activation, preserved BBB integrity, reduced leukocyte infiltration, and suppression of neutrophil pro‐inflammatory signaling within the ischemic brain. Importantly, neutrophil depletion abolished colchicine's protective effects, supporting neutrophils as a major cellular target. Moreover, adjunctive colchicine administration with reperfusion did not alter whole‐blood phagocytosis, bone marrow cellularity, and neutrophil counts at Day 3 after stroke, indicating a relatively safe immunoregulating effect. Ultimately, colchicine reduced brain tissue loss and improved long‐term sensorimotor and cognitive recovery.

In the first experiment of this study, we tested the dose‐range effects of colchicine on the activation of peripheral leukocytes and brain infarction in the tMCAO mice. Our experimental results showed a dose‐dependent suppression of the peripheral neutrophil increase by colchicine, which is associated with a reduction of ischemic brain infarction. The dose‐range effect study also identified colchicine at 0.8 mg/kg as the optimal dose (minimum effective dose) (Figure 1). This dose regimen was selected for investigations of therapeutic effects and underlying pathological mechanisms in the remaining experiments.

Next, we examined whether colchicine modulates early neutrophil responses in the peripheral circulation after tMCAO. tMCAO induced a rapid increase in circulating neutrophils together with an activated phenotype characterized by reduced CD62L, PSGL‐1, and CXCR2 expression. Colchicine blunted the early increase in circulating neutrophils and partially restrained neutrophil activation, as reflected by preservation of CD62L (Figure 2). Circulating neutrophils are among the earliest cellular responders after cerebral ischemia–reperfusion [65], and this early increase may reflect enhanced mobilization from the bone marrow and spleen, as well as altered intravascular margination, tissue redistribution, trafficking, or reduced clearance/cell death [18, 66]. Because CD62L shedding is a rapid and sensitive indicator of early neutrophil priming, its preservation is consistent with reduced neutrophil priming and a lower propensity for firm adhesion and transmigration [67]. By contrast, CXCR2 and PSGL‐1 were not significantly altered at this time point, likely reflecting distinct temporal and regulatory dynamics of these markers during neutrophil activation [68, 69, 70]. In our study, colchicine administration dampens the early systemic neutrophil response after tMCAO, and the resulting reduction in upstream neutrophil availability in the circulation may partly contribute to the attenuation of subsequent thrombo‐inflammatory injury and neuroinflammation.

Next, we validated the pharmacological targeting effects of colchicine in F‐actin polymerization of neutrophils following ischemia–reperfusion. Because it is well known that neutrophil mobilization, activation, degranulation, chemotaxis, adhesion, and NET formation depend on coordinated actin cytoskeletal remodeling [71, 72]. By binding β‐tubulin, colchicine disrupts microtubule dynamics that support actin reorganization, including F‐actin polymerization, thereby broadly suppressing neutrophil effector functions and attenuating neutrophil‐driven thrombo‐inflammation during ischemia–reperfusion [28]. Both phalloidin staining and flow cytometry analysis detected a dramatic increase in F‐actin polymerization in circulating neutrophils after tMCAO, but it was largely reduced by colchicine administration (Figure 3). These findings suggest that colchicine disrupts microtubule–actin crosstalk in neutrophils, limiting actin remodeling required for early inflammatory activation and downstream effector functions [73].

Mechanistically, following reperfusion, neutrophils rapidly adhere to activated endothelium and undergo NET formation, releasing extracellular DNA and proteases that serve as highly thrombogenic scaffolds for platelet aggregation and fibrin deposition [74]. These thrombo‐inflammatory vascular processes are thought to contribute to impaired tissue reperfusion, BBB disruption, and secondary neurovascular injury after reperfusion therapy [14, 75]. Circulating neutrophils showed increased extracellular DNA release as early as 4 h after reperfusion, and this response remained elevated at 24 h. In parallel, intravascular CitH3^+^Ly6G^+^ neutrophils were increased in peri‐infarct cerebral microvessels at 24 h; for this assay, brains were intentionally collected without transcardial perfusion to better preserve intravascular neutrophils and NET‐associated vascular signals [76, 77, 78]. Colchicine significantly reduced intravascular NET‐related signals, together with a marked reduction in intravascular fibrin(ogen) deposition. These findings indicate attenuated NET‐associated thrombo‐inflammation and are consistent with reduced microthrombus‐associated vascular pathology [46]. Moreover, LSCI reflects that colchicine treatment improved regional cortical surface cerebral blood flow at 4 and 24 h after tMCAO, suggesting that its vascular protective effects were associated with improved reperfusion. Together with the early suppression of pathological circulating neutrophil responses, these findings support the possibility that the neurovascular protective effects of colchicine following reperfusion are attributable, at least in part, to attenuation of NET‐related thrombo‐inflammatory vascular pathology.

Besides cerebral microvascular patency impairment, another important complication of ischemia–reperfusion therapy is cerebrovascular inflammation and BBB disruption [79, 80]. In vivo, colchicine reduced endothelial adhesion molecule expression, decreased IgG extravasation, and partially restored tight‐junction‐related transcripts after tMCAO. In contrast, in an in vitro hypoxia/reoxygenation plus IL‐1β model, colchicine did not improve endothelial permeability or restore ZO‐1 expression, although it modestly suppressed adhesion molecule expression (Figure 5). These findings suggest that colchicine does not exert a strong direct barrier‐protective effect on endothelial cells in vitro but may partially modulate endothelial inflammatory activation [81, 82]. Thus, the BBB protection observed in vivo is more likely driven predominantly by indirect mechanisms, particularly suppression of neutrophil‐mediated thrombo‐inflammatory injury to the cerebrovascular endothelium. A contributory direct endothelial effect cannot be excluded, but the mechanism underlying reduced adhesion molecule expression was not resolved in this study and may involve cytoskeletal reorganization, altered inflammatory signaling, transcriptional regulation, or a combination of these processes [82, 83]. All these important questions need to be clearly defined in the next step.

Because leukocyte infiltration and neutrophil‐derived inflammatory signaling are major drivers of secondary brain injury after ischemic stroke [84], we further examined whether colchicine modulates immune cell recruitment and neutrophil activation within the brain after tMCAO. At 24 h post‐tMCAO, colchicine markedly reduced brain infiltration of neutrophils, Ly6C^high^ monocytes, and B cells, whereas also decreasing microglial accumulation, without affecting T cell or NK cell numbers. Importantly, colchicine significantly suppressed IL‐1β production in brain‐infiltrating neutrophils, indicating attenuation of local neutrophil pro‐inflammatory activation (Figure 6). These findings demonstrate that colchicine dampens post‐ischemic neuroinflammation by limiting both leukocyte recruitment and neutrophil effector responses within the injured brain. Mechanistically, colchicine's inhibition of neutrophil cytoskeletal remodeling likely restrains early neutrophil activation, adhesion, and transmigration, thereby secondarily reducing recruitment of other myeloid populations and microglial response [71, 85, 86]. Although we cannot exclude additional direct effects of colchicine on other immune or resident glial populations, our data are most consistent with a neutrophil‐centered mechanism in which blunting pathological neutrophil activation contributes to downstream reductions in neuroinflammatory cell recruitment and microglial accumulation. Notably, colchicine did not reduce T cell or NK cell numbers in the brain at 24 h, suggesting that its neuroprotection is not accompanied by overt suppression of lymphocyte infiltration at this time point. By selectively targeting pathological neutrophil responses without broadly suppressing lymphocyte infiltration, colchicine uncouples neuroprotection from generalized immunosuppression [64]. These findings support further evaluation of colchicine as an adjunctive therapy to limit secondary neuroinflammation and tissue damage following reperfusion in ischemic stroke.

It has been known that post‐stroke immunosuppression contributes to infection susceptibility and poor clinical outcomes [64]. It was important to assess whether colchicine alters systemic innate immune function after stroke. Although tMCAO reduced the phagocytic capacity of circulating neutrophils and F4/80^+^ monocytes, and bone marrow cellularity and neutrophil counts at Day 3 after stroke, no further reduction was found in the colchicine‐treated group (Figure 7). These experimental findings suggest that colchicine's neurovascular protective effects are not accompanied by additional post‐stroke innate immune suppression. Instead, colchicine appears to selectively restrain early pathological neutrophil activation during reperfusion without further impairing innate immune function, suggesting a translational potential of colchicine as an adjunct to reperfusion interventions in acute ischemic stroke.

Next, we evaluated whether colchicine's acute neurovascular protection translates into sustained neurological benefit after tMCAO. This is because early thrombo‐inflammatory injury and microvascular dysfunction after reperfusion are major determinants of delayed neuronal loss and long‐term functional outcome [14]. Our experimental results showed colchicine treatment at reperfusion significantly improved sensorimotor performance across multiple behavioral paradigms, including mNSS, foot‐fault, and adhesive removal tests, and enhanced spatial reference memory in the Y‐maze at 14 days post‐stroke. Consistent with these functional improvements, colchicine markedly reduced chronic brain tissue loss assessed by MAP2 staining (Figure 8). These findings indicate that transient colchicine administration during the reperfusion phase confers durable neuroprotection and promotes long‐term functional recovery. Importantly, the sustained benefits observed at 2 weeks indicate a durable protective effect rather than a transient improvement limited to the acute phase. By mitigating early neutrophil‐driven thrombo‐inflammation and secondary neurovascular injury, colchicine may limit progressive tissue degeneration and thereby improve long‐term recovery. Together, these results support the translational relevance of targeting early inflammatory cytoskeletal mechanisms, which can yield lasting structural and functional benefits after ischemic stroke, supporting colchicine as a promising adjunct to reperfusion therapy.

Another major concern is related to potential off‐target effects. Because colchicine has pleiotropic anti‐inflammatory properties, we sought to determine whether its neuroprotective effects after tMCAO involve neutrophil‐independent mechanisms. To directly test neutrophil dependence, we depleted circulating neutrophils using an anti‐Ly6G antibody before and after stroke induction. Effective depletion (> 90%) significantly reduced ischemic infarct size and improved neurological outcomes, consistent with prior studies [18]. Under circulating neutrophil depletion, colchicine no longer conferred additional protection, supporting that neutrophils are a major cell population of colchicine in this model. However, anti‐Ly6G‐mediated neutrophil depletion is a broad systemic intervention and may alter the inflammatory milieu beyond neutrophils alone [87, 88, 89]. In addition, because neutrophils are functionally coupled to platelets, endothelial activation, and microglial responses during post‐stroke thrombo‐inflammation, systemic neutrophil depletion may also indirectly reshape these compartments and thereby mask potential additional effects of colchicine on other components of the neurovascular unit [66, 90, 91]. Therefore, our findings support a predominantly neutrophil‐dependent mechanism. However, in the next step, it would be important to distinguish primary neutrophil‐mediated effects from secondary changes in other vascular and immune cell components [89, 92] using more selective strategies including partial depletion approaches and neutrophil‐specific genetic models.

We are aware of several limitations of the present study. First, the intraluminal filament tMCAO model provides controlled ischemia followed by abrupt reperfusion, but it does not reproduce key procedural aspects of clinical mechanical thrombectomy including thrombus‐device interaction, microcatheter manipulation, clot retrieval, distal embolization, or procedure‐related vascular injury and no‐flow phenomenon. Accordingly, this model was used to examine the pathological changes and underlying mechanisms associated with thrombo‐inflammation and microvascular dysfunction after recanalization [93]. Second, all experiments were conducted in young, healthy male mice. Although this design enabled mechanistic investigation in a controlled tMCAO/reperfusion setting, it does not capture the heterogeneity of clinical ischemic stroke populations. Therefore, our findings provide initial preclinical mechanistic evidence. Future studies in aged animals, both sexes, and models with relevant comorbidities will be necessary to better assess the translational potential of colchicine as an adjunct to reperfusion therapy. Third, colchicine was administered as a single dose at reperfusion to target the early phase of neutrophil‐driven thrombo‐inflammatory responses and microvascular injury. However, this design does not define the full therapeutic window, as it does not address the effects of pre‐reperfusion treatment, delayed post‐reperfusion administration, or repeated dosing. Accordingly, the translational relevance of this dosing paradigm should be interpreted with caution. Future studies examining alternative timing and dosing strategies will be necessary to better define the therapeutic window of colchicine. Fourth, because of the exploratory nature of this study and the large number of endpoints examined, no formal study‐wide adjustment for multiple comparisons was performed. Additional preclinical studies will be needed to further validate these findings. Nevertheless, the main conclusions are supported by consistent findings across multiple complementary outcome measures rather than by isolated endpoints. Fifth, although neutrophil depletion provides strong evidence that colchicine's neuroprotective effects are neutrophil dependent, the precise cytoskeletal regulators and NET‐associated pathways responsible for this protection were not genetically dissected. At last, while acute innate immune function was preserved, potential delayed or cumulative immune effects beyond the subacute phase were not systematically evaluated. Additional studies should therefore validate the efficacy of colchicine in both male and female, aged, and comorbid stroke models and perform comprehensive pharmacokinetic and pharmacodynamic analyses to optimize dosing and timing. Cell‐specific genetic approaches targeting neutrophil cytoskeletal remodeling and NET formation will be essential to establish causal mechanisms. Finally, evaluating colchicine in combination with intravenous tPA or mechanical thrombectomy, together with the development of circulating NET or microvascular perfusion biomarkers, will be critical to enhance translational relevance and inform the design of early‐phase clinical trials.

## 5 Conclusion and Future Translational Perspective

Together, our findings highlight a critical role for neutrophil cytoskeletal regulation and NET formation–driven microvascular thrombo‐inflammation in post‐reperfusion microvascular dysfunction, BBB disruption, and secondary brain injury. By acting as a pan‐cytoskeletal brake on pathological neutrophil responses, colchicine may help uncouple large‐vessel recanalization from microvascular failure and neurovascular injury. These results provide strong mechanistic and functional evidence supporting colchicine as a potential adjunct to reperfusion therapy in acute ischemic stroke.

Given colchicine's established clinical use and favorable safety profile, these findings indicate a potential translational relevance. A small‐scale early‐phase exploratory clinical study might be considered in patients undergoing mechanical thrombectomy, with or without intravenous thrombolysis, to assess the related biomarker responses and initially evaluate the adjunctive therapeutic potential. However, clinical feasibility will require defining a workflow‐compatible dosing window around reperfusion without delaying standard treatment. Future preclinical studies should define optimal dosing and timing relative to reperfusion therapy, assess efficacy in aged and comorbid animal models, and evaluate circulating NET‐related markers and neutrophil activation markers such as CD62L expression to guide patient stratification and pharmacodynamic monitoring. Ultimately, targeting neutrophil cytoskeletal dynamics might be developed as a broadly applicable strategy to improve tissue‐level reperfusion and long‐term outcomes in stroke patients undergoing reperfusion therapy.

## Author Contributions

X.W. and Q.L. formulated the concept and designed the studies. D.W., Y.X., M.S., Y.W., and D.Z. performed the experiments. D.W., Y.X., M.D.K., T.Y.S., Y.J., L.D., N.K., and N.L. analyzed the results. D.W., A.S.D., Q.L., and X.W. interpreted the results and drafted the manuscript.

## Funding

This work was supported in part by the American Heart Association through a Postdoctoral Fellowship (23POST1026423 to Yinghua Jiang), a Career Development Award (23CDA1055341 to Ning Liu), and a Predoctoral Fellowship (26PRE1560109 to Yingjie Wang).

## Ethics Statement

All animal experiments were performed with ethics approval from the Institutional Animal Care and Use Committee (IACUC) of Tulane University (Protocol ID: 2197).

## Consent

The authors have nothing to report.

## Conflicts of Interest

The authors declare no conflicts of interest.

## Supporting information


**Figures S1–S6:** cns70922‐sup‐0001‐FiguresS1‐S6.docx.

## Data Availability

The data that support the findings of this study are available from the corresponding author upon reasonable request.
