# Supplementary material for: Colchicine as an Adjunctive Therapy to Improve Ischemic Stroke Reperfusion Outcomes in Mice
Source: CNS Neurosci Ther. 2026 Jul 9;32(7):e70922. doi: 10.1002/cns.70922 (PMC13349406; doi:10.1002/cns.70922)
Supplement: Supplementary file 1 — Figures S1–S6: cns70922‐sup‐0001‐FiguresS1‐S6.docx. [file CNS-32-e70922-s001.docx]

**Supplemental Materials**

**Colchicine as an Adjunctive Therapy to Improve Ischemic Stroke Reperfusion Outcomes in Mice**

Danni Wang^1,2†^, Yuwen Xiu^2†^, Mengxuan Shi^2^, Yingjie Wang^2^, Di Zhou^2^, Mitchell D Kilgore^2^, Lauren Dumont^2^, Thin Yadanar Sein^2^, Yinghua Jiang^2^, Nicole Kazour^2^, Ning Liu^2^, Aaron S. Dumont^2^, Qiang Liu^1*^, Xiaoying Wang^2*^

1. Department of Neurology, Tianjin Neurological Institute, Tianjin Medical University General Hospital, Tianjin 300052, China.

2. Clinical Neuroscience Research Center, Department of Neurosurgery and Neurology, Tulane University School of Medicine, New Orleans, LA 70112, USA.

***Correspondence Author:**

Qiang Liu, MD, PhD, Department of Neurology, Tianjin Neurological Institute, Tianjin Medical University General Hospital, Tianjin 300052, China. Email address: qliu@tmu.edu.cn

or

Xiaoying Wang, MD, PhD, Clinical Neuroscience Research Center, Department of Neurosurgery and Neurology, Tulane University School of Medicine, New Orleans, LA 70112, USA. Email address: [xwang51@tulane.edu](mailto:xwang51@tulane.edu)

†These authors contributed equally to this work.

**Supplemental Methods**

**Flow cytometry**

At 4 h, 24 h, or 3 d after tMCAO, mice were euthanized under deep isoflurane anesthesia, and peripheral blood, brain, spleen, and femur/bone marrow were collected for flow cytometric analysis, as previously described [41,42]. Peripheral blood was first collected into EDTA-containing tubes. Spleens and femurs were subsequently harvested. For brain immune cell isolation, mice were then transcardially perfused with cold PBS until the liver became pale, after which the brain was rapidly removed.

For spleen cell isolation, spleens were mechanically dissociated in PBS to generate single-cell suspensions and passed through a cell strainer. Cells were centrifuged at 330 × g for 5 min at 4 °C, resuspended in 1× RBC lysis buffer (BioLegend, 420301), and incubated for 10 min at room temperature in the dark. Lysis was stopped by adding PBS. Cells were then centrifuged again at 330 × g for 5 min at 4 °C, washed as needed, and then resuspended for antibody staining.

Peripheral blood samples were processed by adding blood into FACS tubes containing 2% FBS (ThermoFisher, A5669801), followed by the antibody mix. After surface staining at 4 °C for 30 minutes, 1× RBC lysis buffer was added per tube and incubated for 10 min at room temperature in the dark. Lysis was terminated with PBS, followed by centrifugation at 330 × g for 5 min at 4 °C. Cells were then resuspended in 2% FBS for counting and subsequent analysis.

For brain cell isolation, mice were transcardially perfused with cold PBS until the liver became pale. The ipsilateral hemisphere was minced in collagenase I (1 mg/mL) and incubated at 37 °C for 30 min with intermittent mixing. Digestion was stopped by adding PBS, and the suspension was transferred to a centrifuge tube. After centrifugation at 492 × g for 5 min, the pellet was resuspended in 30% Percoll (Fisher Scientific, 45-001-747) and centrifuged at 700 × g for 10 min at 4 °C with low brake to remove myelin and debris. The resulting cell pellet was resuspended in PBS. Cells were then counted, pelleted again, and finally resuspended in 2% FBS for staining.

For bone marrow cell isolation, both ends of the femur were cut, and bone marrow was flushed out using 300–500 μL 2% FBS with a syringe, yielding a final volume of approximately 1 mL. The suspension was gently dispersed, treated with 1× RBC lysis buffer, and incubated for 10 min at room temperature in the dark. Lysis was stopped with PBS, followed by centrifugation at 330 × g for 5 min at 4 °C. Cells were resuspended in 2% FBS, counted, and aliquoted into FACS tubes for staining.

The obtained single-cell suspensions were co-stained with antibodies at 4 °C for 30 minutes. For intracellular staining, cells were fixed and permeabilized using the intracellular fixation and permeabilization buffer set (Biolegend, 426803). The following antibodies were used: CD3 (Biolegend, 100204), CD19 (Invitrogen, 366-0193-82), CD11b (Biolegend, 101206), CD45 (Biolegend, 103112), Ly6C ( Biolegend, 128016), Ly6G (Biolegend, 127622), CXCR2 (Biolegend, 149312), NK1.1 (Invitrogen, 25-5941-82), CD162 (Invitrogen, 46-1621-80), CD62L (Biolegend, 104410), SYTOX Green (Invitrogen, S7020), Phalloilldin (Proteintech, PF00001), IL-1β (Invitrogen, 12-7114-82), TNF-α (Biolegend, 506304), IL-6 (Biolegend, 504504). The data were acquired by a BD LSRFortessa^TM^ Cell Analyzer (BD Bioscience) and analyzed with FlowJo software (Version 10.9.0, FlowJo LLC).

**Immunostaining**

Immunostaining was conducted as we previously described [45]. To trace blood vessels, mice received an intravenous injection of fluorescein-labeled Lycopersicon esculentum (tomato) lectin (Vector Laboratories, DL-1177-1/DL-1178-1; 1.25 mg/kg) 5 min before sacrifice at 24 h after tMCAO. For intravascular neutrophils/NETs, brains were collected without transcardial perfusion to preserve luminal and loosely adherent signals [46], embedded in OCT, snap-frozen, and cryosectioned (10 μm coronal sections; Leica CM1950). Sections were fixed in 4% paraformaldehyde and blocked/permeabilized (5% donkey serum, 3% BSA, 0.1% Triton X-100), then incubated overnight at 4°C with rat anti-mouse Ly6G (BD, 551459) and anti-citrullinated histone H3 (Abcam, ab5103), followed by donkey anti-rat AF488 (Invitrogen A21208) and donkey anti-rabbit AF594 (Invitrogen A32754) for 2 h at room temperature. For fibrin(ogen) deposition and endogenous IgG extravasation, mice were perfused with ice-cold PBS to minimize intravascular background. Brains were fixed in 4% paraformaldehyde, cryoprotected in 15%/30% sucrose, embedded in OCT, and sectioned (8 μm). Sections were stained with anti-fibrinogen γ chain (Abcam, ab281924) followed by donkey anti-rabbit AF488 (Invitrogen, A21206). BBB leakage was assessed by staining endogenous mouse IgG with donkey anti-mouse IgG AF488 (Invitrogen, A21202) for 1 hour at room temperature. Slides were mounted with DAPI-containing antifade medium (Vector, H-1500-10). Images were acquired on a confocal microscope using identical settings within each experiment and analyzed in ImageJ using a predefined threshold applied uniformly to all images within the same analysis batch [42]. For each mouse, three peri-infarct coronal sections spaced 300 μm apart were analyzed, with three non-overlapping fields per section, and values were averaged to generate one measurement per mouse for statistical analysis. Within tomato lectin-labeled vessels, intravascular CitH3⁺ Ly6G⁺ neutrophils were counted. Fibrin(ogen) deposition was quantified as the positive area fraction within vascular regions, and BBB leakage was quantified as the IgG-positive area fraction in the peri-infarct region [42,45]. No additional normalization across samples was applied beyond identical image acquisition settings and uniform analysis parameters within the same experiment.

**Cell culture and hypoxia/reoxygenation (H/R) treatment**

Primary human brain microvascular endothelial cells (HBMVECs) were purchased from Cell Systems and cultured in EBM-2 (Lonza, CC-3156) with growth supplements. To mimic the in vivo environment of ischemia–reperfusion after stroke, we applied a cell injury model using hypoxia plus IL-1β followed by reoxygenation treatment in cultured HBMVECs as we previously described [55,56]. Briefly, when the cells were cultured to ~100% confluence, EBM-2 medium was replaced with serum-free DMEM (Fisher Scientific, 11320082). Monolayers were exposed to IL-1β (20 ng/mL; Thermofisher, 200-01B-50UG) and subjected to 4 h hypoxia in a Billups-Rothenberg chamber, followed by reoxygenation with colchicine (35 nM) or vehicle added at the onset of reoxygenation and incubation under normoxia for 20 h. The 35 nM colchicine dose was selected to approximate the estimated mean 0–24 h plasma exposure after a single 0.8 mg/kg i.p. dose in mice, based on standard noncompartmental pharmacokinetic relationships and prior mouse PK data [57,58].

**In vitro trans-endothelial permeability assay**

Trans-endothelial permeability was assessed by quantifying FITC–dextran flux across confluent endothelial monolayers as we previously described [55,59]. Briefly, primary HBMVECs were cultured on the inner surface of collagen-coated Transwell inserts (12 mm, 0.4 µm; Corning). After treating each group of HBMVECs monolayers with different conditions, FITC–dextran (0.1 mg/mL; Sigma-Aldrich, 46954) was then added to the apical chamber and, after 60 min, fluorescence in the basolateral chamber was measured (Ex 490 nm/Em 520 nm). Experiments were repeated independently with triplicate technical replicates

**Cell Viability Assay**

The viability of cultured endothelial cells was assessed using the 3-(4,5-dimethylthiazol-2-yl)- 2,5-diphenyltetrazolium bromide (MTT) reduction assay [60]. Briefly, after treatment, HBMVEC were placed in media containing 0.4% MTT (Thermo Fisher, M6494). After 3 hours at 37°C, the media was removed, and cells were dissolved in DMSO. Formazan formation was measured by reading absorbance at 570 nm with a reference setting of 630 nm on a microplate reader (Infinite M Plex, TECAN). Experiments were repeated independently with triplicate technical replicates

**Real-time quantitative PCR analysis**

For mouse samples, Real-time quantitative PCR (RT-qPCR) was performed as we described previously [42]. At 24 h after tMCAO, total RNA was isolated from mouse brain microvessels using the miRNeasy Micro Kit (Qiagen, 217084) and reverse-transcribed using the QuantiTect Reverse Transcription Kit (Qiagen, 205311). qPCR was performed using TaqMan Fast Advanced Master Mix (Applied Biosystems, 4444963) and analyzed on an ABI 7500 Real-Time PCR system (Applied Biosystems) in technical triplicate. Relative expression was calculated using the 2^−ΔΔCt method with normalization to the indicated housekeeping gene. Probes (Thermo Fisher Scientific) included Hprt (Mm01545399_m1), Icam1 (Mm00516023_m1), Vcam1 (Mm01320970_m1), Sele (Mm00441278_m1), Selp (Mm01295931_m1), Cldn5 (Mm00727012_s1), Ocln (Mm00500910_m1), and Tjp1 (Mm01320638_m1).

For HBMVECs, total RNA was extracted using the miRNeasy Micro Kit (Qiagen) and reverse-transcribed using the QuantiTect Reverse Transcription Kit (Qiagen, 205311) [43]. qPCR was performed using TaqMan Fast Advanced Master Mix (Applied Biosystems, 4444963) and analyzed on an ABI 7500 Real-Time PCR system (Applied Biosystems) in technical triplicate. Human probes were TJP1/ZO-1 (Hs01551861_m1), VCAM1 (Hs01003372_m1), and ICAM1 (Hs00164932_m1), with GAPDH (Hs02758991_g1) as the endogenous control. Relative expression was calculated using the 2^−ΔΔCt method with normalization to the indicated housekeeping gene.

**Supplemental Figure 1
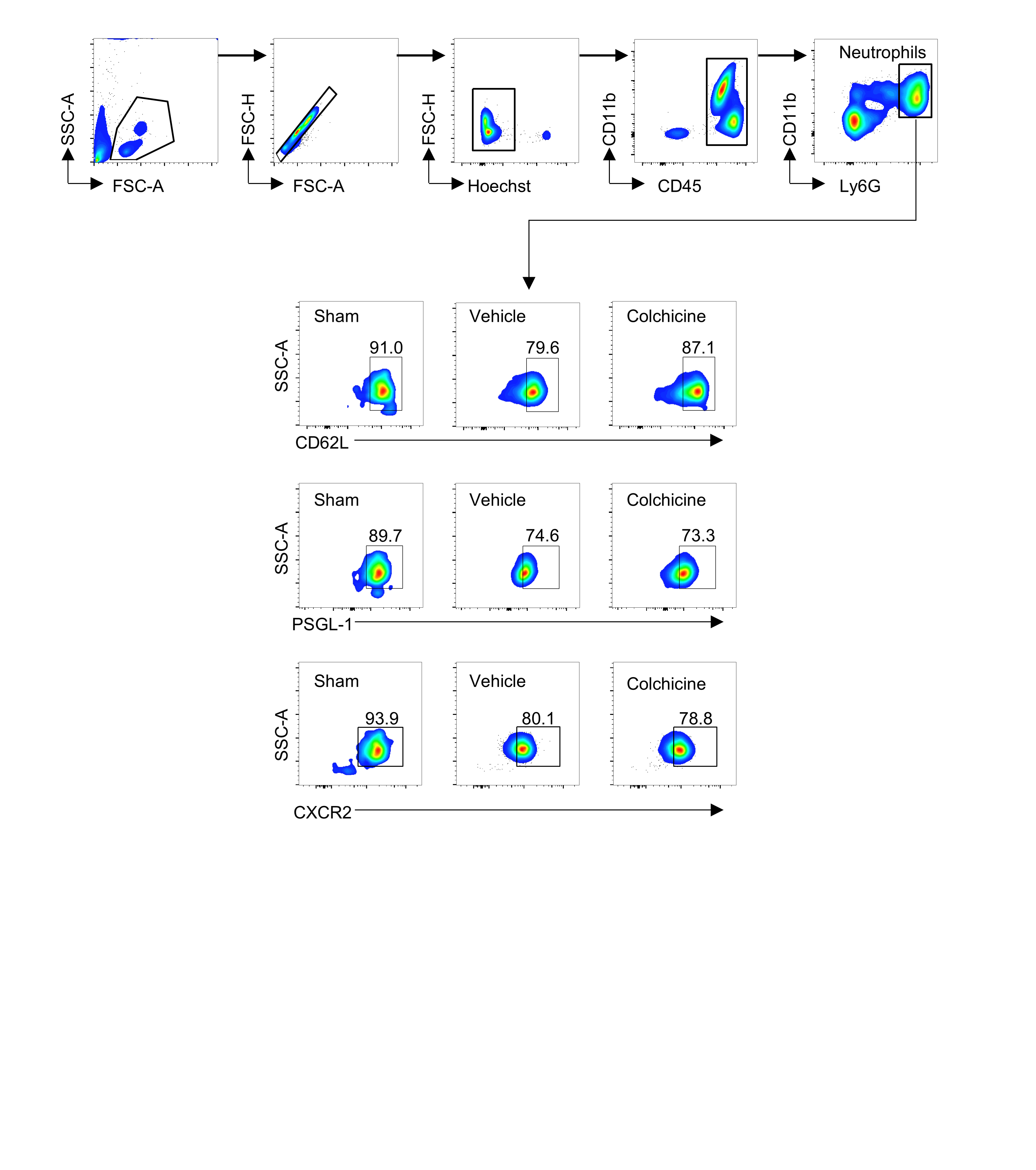
**

**Supplemental Figure 1.** Full sequential gating strategy for peripheral blood neutrophil activation marker (CD62L, PSGL-1, and CXCR2) analysis.

**Supplemental Figure 2
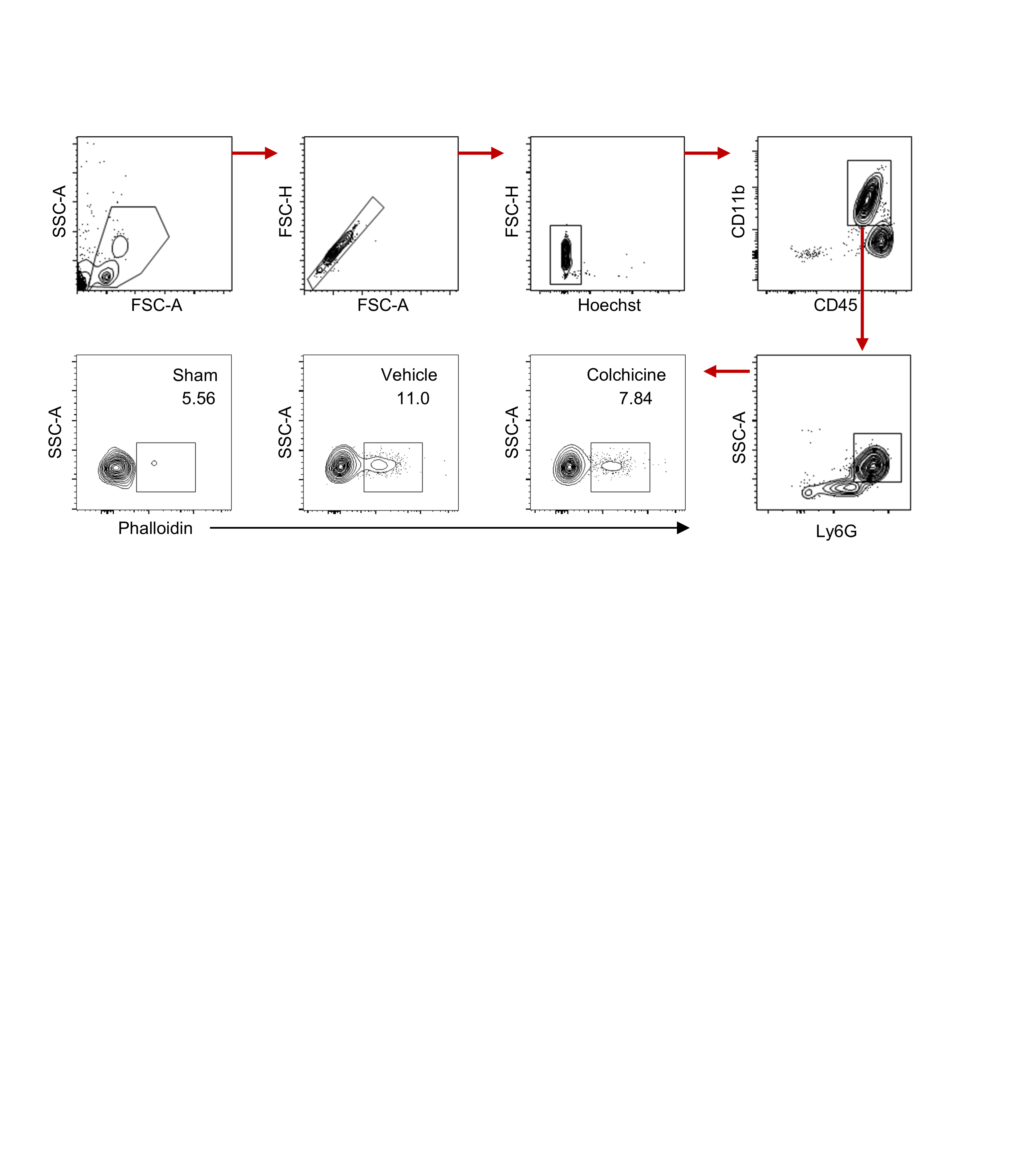
**

**Supplemental Figure 2.** Full sequential gating strategy for peripheral blood neutrophil phalloidin/F-actin analysis.

**Supplemental Figure 3
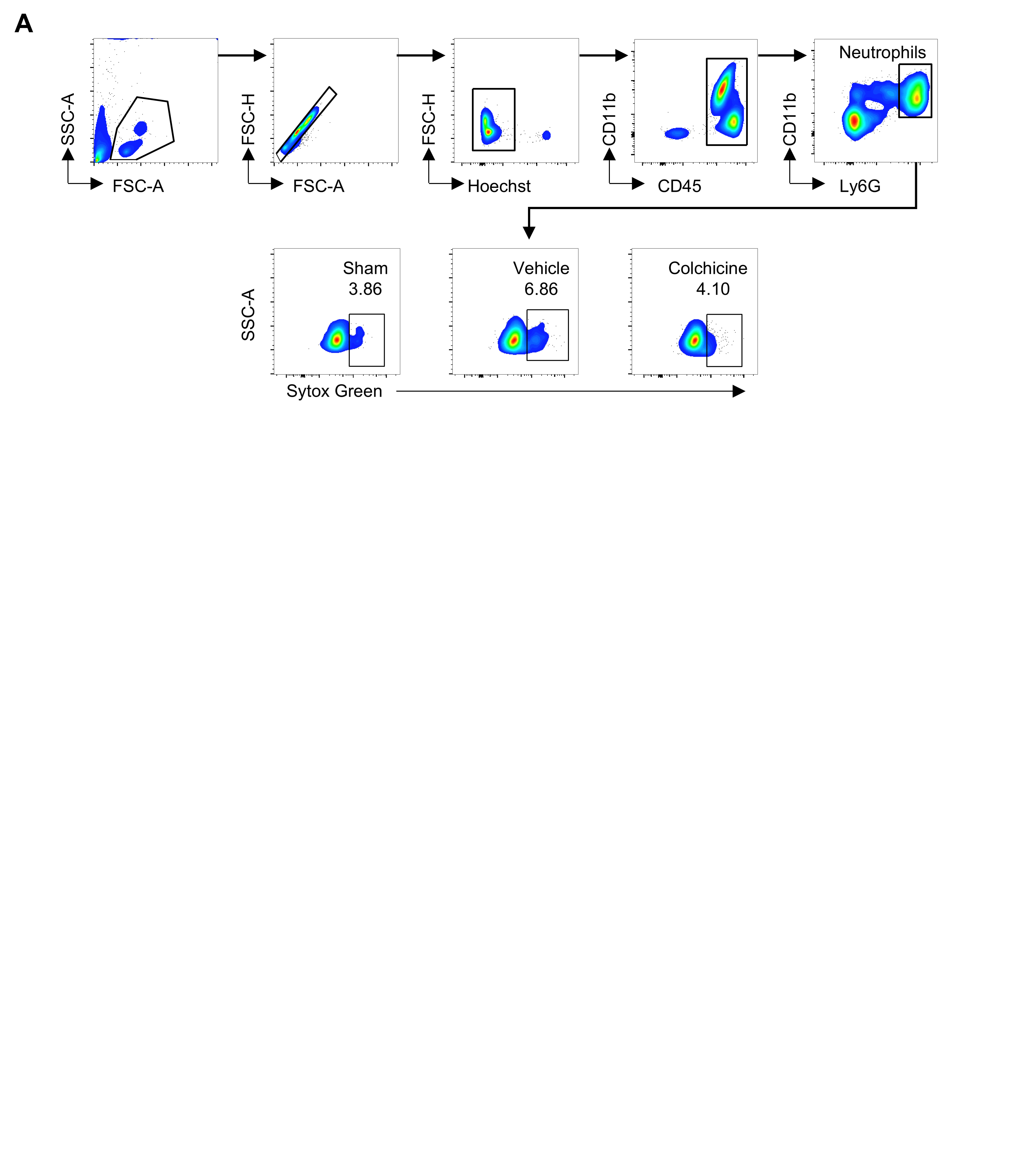
**

**Supplemental Figure 3.** Full sequential gating strategy for peripheral blood neutrophil SYTOX Green analysis.

**Supplemental Figure 4
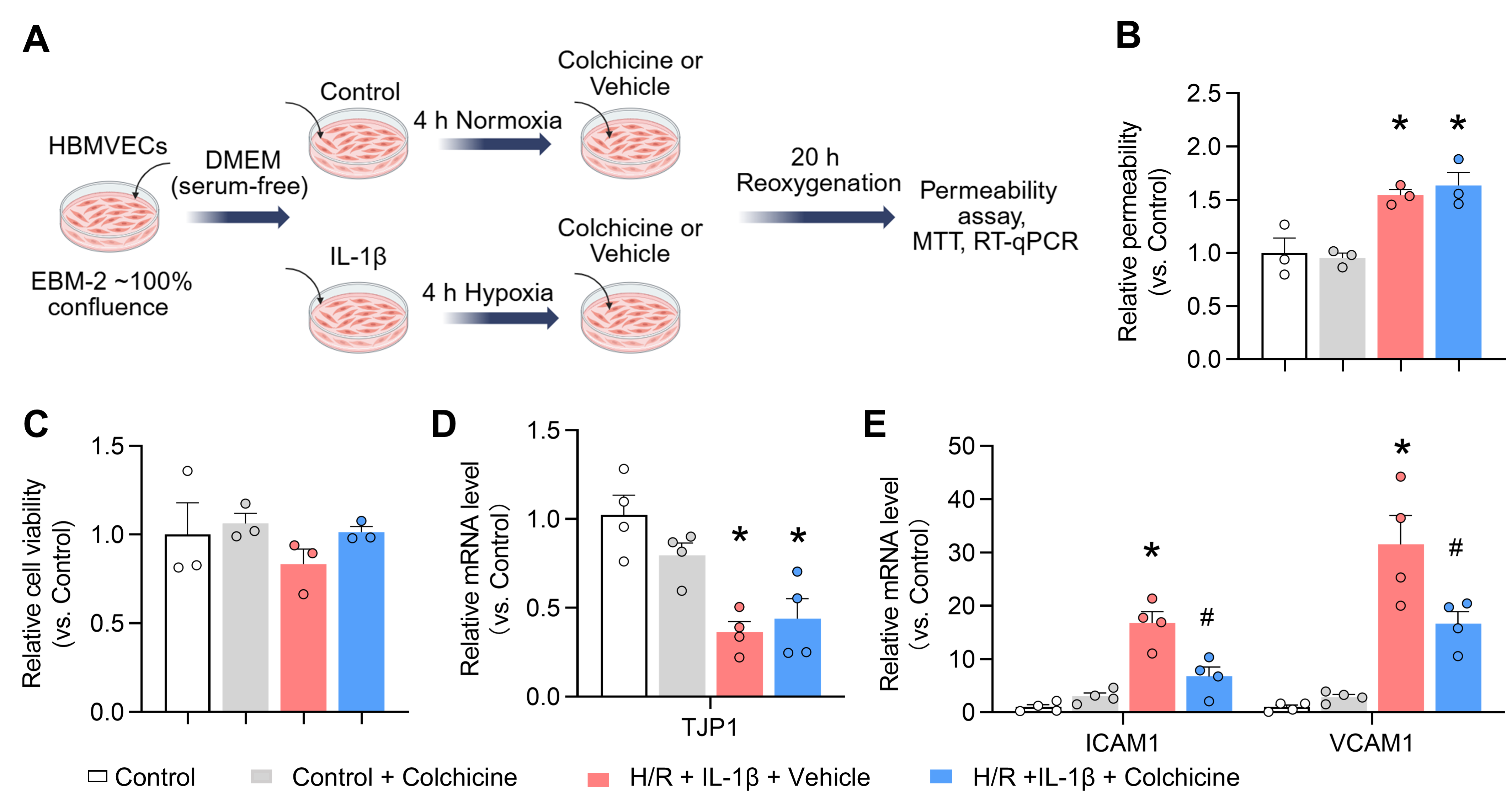
**

**Supplemental Figure 4.** Colchicine has no direct effect on trans-endothelial permeability in an in vitro BBB injury model. HBMVEC monolayers were exposed to hypoxia for 4 h in the presence of IL-1β (20 ng/mL), followed by 20 h reoxygenation; colchicine (35 nM) or vehicle (PBS) was added at the onset of reoxygenation. (**A**). Schematic diagram of the experimental design. **(B).** Relative fold change of FITC-dextran permeability in cultured HBMVECs. Four independent experiments were performed, with n=3 sister cultures per experiment. **(C)** Cell viability after various treatments was assessed using the MTT assay. Four independent experiments, with n=3 sister cultures per experiment. **(D-E)** RT–qPCR analysis of TJP1, ICAM, and VCAM1 mRNA levels in HBMVECs. Three independent experiments, with n = 4 sister cultures per experiment. Mean ± SEM; *P < 0.05 vs. Control; #P < 0.05 vs. H/R + IL-1β + Vehicle. One-way ANOVA.

**Supplemental Figure 5
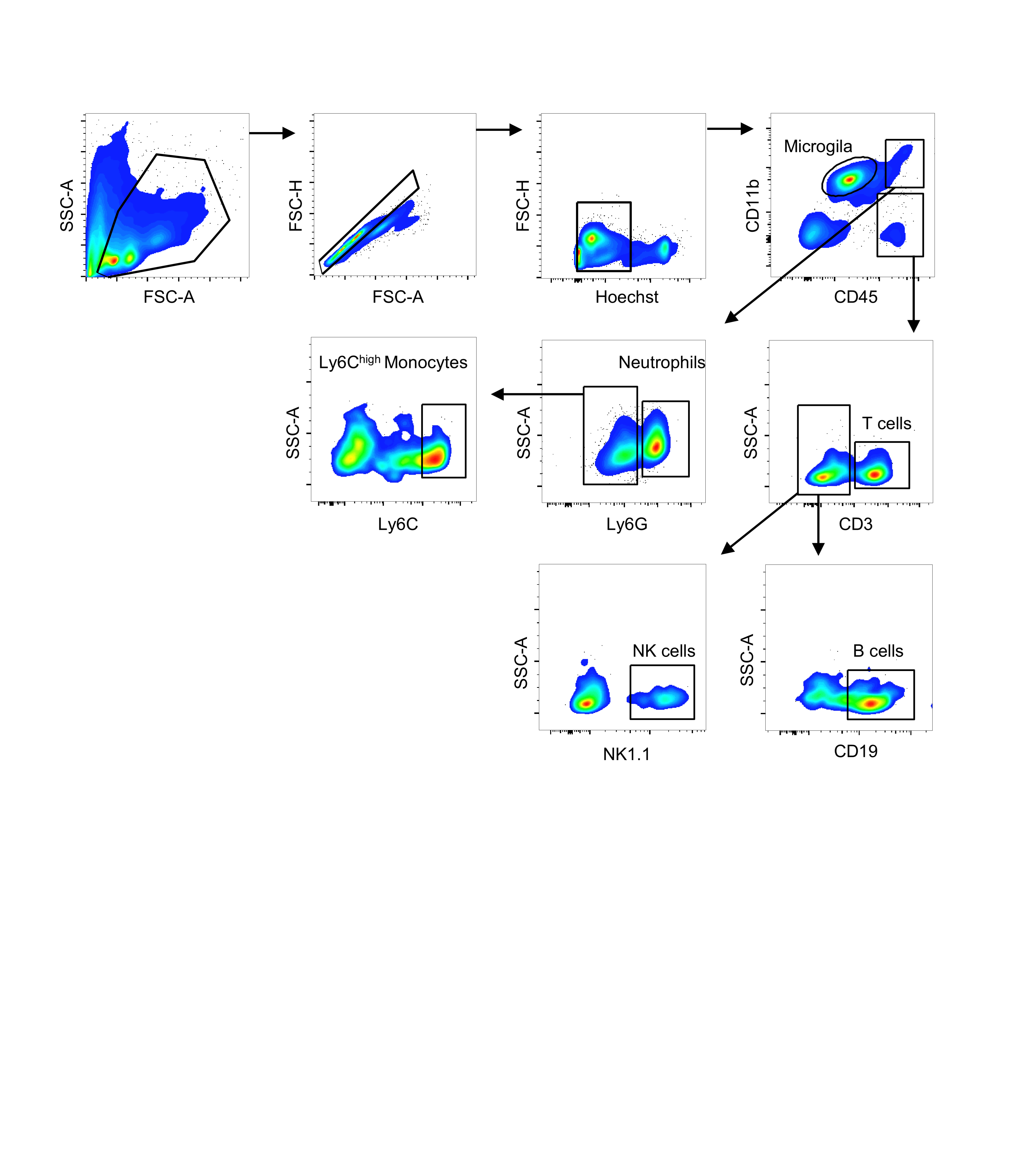
**

**Supplemental Figure 5.** Full sequential gating strategy for brain immune cell subset analysis. Neutrophils (CD11b^+^ CD45^high^ Ly6G^+^), Ly6C ^high^ Monocytes (CD11b^+^ CD45^high^ Ly6G^-^ Ly6C^high^), T cells (CD11b^-^ CD45^high^ CD3^+^), B cells (CD11b^-^ CD45^high^ CD19^+^), NK cells (CD45^high^ CD3^-^ NK1.1^+^), and microglia (CD45^int^ CD11b^+^).

**Supplemental Figure 6
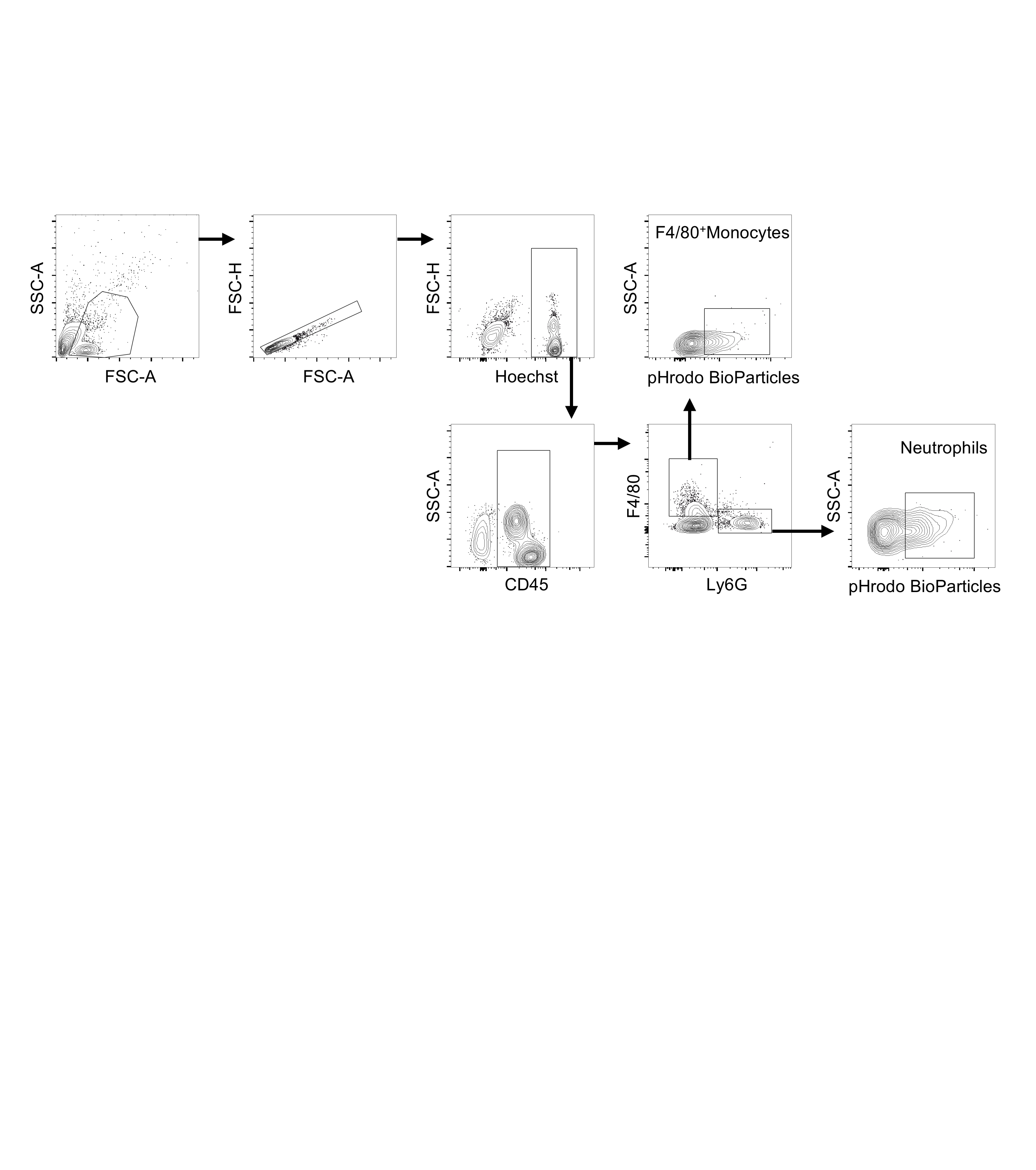
**

**Supplemental Figure 6.** Full sequential gating strategy for the blood phagocytosis assay. Neutrophils (CD45^+^ Ly6G^+^), F4/80⁺ monocytes (CD45^+^ F4/80^+^).
